# Modelling how responsiveness to interferon improves interferon-free treatment of hepatitis C virus infection

**DOI:** 10.1371/journal.pcbi.1006335

**Published:** 2018-07-12

**Authors:** Vishnu Venugopal, Pranesh Padmanabhan, Rubesh Raja, Narendra M. Dixit

**Affiliations:** 1 Department of Chemical Engineering, Indian Institute of Science, Bangalore, India; 2 Centre for Biosystems Science and Engineering, Indian Institute of Science, Bangalore, India; ETH Zurich, SWITZERLAND

## Abstract

Direct-acting antiviral agents (DAAs) for hepatitis C treatment tend to fare better in individuals who are also likely to respond well to interferon-alpha (IFN), a surprising correlation given that DAAs target specific viral proteins whereas IFN triggers a generic antiviral immune response. Here, we posit a causal relationship between IFN-responsiveness and DAA treatment outcome. IFN-responsiveness restricts viral replication, which would prevent the growth of viral variants resistant to DAAs and improve treatment outcome. To test this hypothesis, we developed a multiscale mathematical model integrating IFN-responsiveness at the cellular level, viral kinetics and evolution leading to drug resistance at the individual level, and treatment outcome at the population level. Model predictions quantitatively captured data from over 50 clinical trials demonstrating poorer response to DAAs in previous non-responders to IFN than treatment-naïve individuals, presenting strong evidence supporting the hypothesis. Model predictions additionally described several unexplained clinical observations, viz., the percentages of infected individuals who 1) spontaneously clear HCV, 2) get chronically infected but respond to IFN-based therapy, and 3) fail IFN-based therapy but respond to DAA-based therapy, resulting in a comprehensive understanding of HCV infection and treatment. An implication of the causal relationship is that failure of DAA-based treatments may be averted by adding IFN, a strategy of potential use in settings with limited access to DAAs. A second, wider implication is that individuals with greater IFN-responsiveness would require shorter DAA-based treatment durations, presenting a basis and a promising population for response-guided therapy.

## Author summary

Treatment of hepatitis C virus (HCV) infection is seeing a paradigm shift with powerful drugs called direct-acting antiviral agents (DAAs) replacing earlier treatments involving interferon (IFN). DAAs target specific HCV proteins. IFN stimulates our immune response against HCV. The two should thus work independently. Surprisingly, DAAs appear to work better in individuals who also tend to respond well to IFN. We hypothesized here that responsiveness to DAAs and IFN are causally linked. IFN can suppress viral replication, preventing the development of resistance to DAAs and improve DAA treatments. Using a new multiscale mathematical model and analysis of a vast body of clinical data, we found strong evidence supporting this hypothesis. Leveraging the causal relationship, we suggest new ways of optimizing DAA treatments, potentially improving their efficacy, tolerability, affordability and access.

## Introduction

Direct-acting antiviral agents (DAAs) are revolutionizing the treatment of chronic hepatitis C virus (HCV) infection. Sustained virological response (SVR) rates of over 90% have been achieved in recent clinical trials with all-oral DAA treatments lasting as short as 12 weeks, in striking contrast to the combination of pegylated interferon and ribavirin (PR), which elicited SVR rates of only ~50% with 24–48 weeks of treatment [1, 2]. Indeed, DAAs are rapidly replacing PR as the treatment of choice for chronic HCV infection [2]. An intriguing feature of DAAs is the differential response they elicit in individuals who respond differently to PR: They seem to work better in individuals who also tend to be more responsive to PR. For instance, with the combination of the DAAs ledipasvir and sofosbuvir, SVR rates dropped from nearly 100% in treatment-naive individuals to ~87% in PR-experienced individuals infected with HCV genotype 1b [1]. This differential response appears more significant with the older generation of DAAs than the newer ones, but is evident across clinical trials and across DAAs (Table 1). Treatment guidelines for those who previously failed PR treatment are different from treatment naïve patients [3]. Interferon (IFN) acts primarily by stimulating the innate immune response to HCV [4]. Ribavirin is thought to potentiate the activity of IFN [5, 6]. DAAs, on the other hand, target specific HCV proteins, independently of host immune responses [7]. Why responsiveness to IFN should improve outcomes of DAA-based treatments is thus puzzling.

**Table 1:** Response to DAA-based treatments. SVR rates elicited by various IFN-free and IFN-containing DAA combinations in treatment-naïve and prior null responders to PR from recent clinical trials. The treated population size is indicated in brackets. The significance of the difference in the SVR rates in the two populations is computed using the χ^2^ test. The HCV genotype and whether the patients had liver cirrhosis is indicated. The details of the treatment regimens along with the sources of the data are in S1 Table.

	Regimen	Genotype	Cirrhosis	% SVR (N)		P-value
				Naïve	Null	
IFN-containing regimen	Telaprevir + PR	1	nd^$^	75.4 (1272)	32 (147)	5.2×10^−28^
		1	no	68.7 (941)	50.7 (213)	6.7×10^−7^
		1	yes	45.4 (291)	26.6 (79)	2.6×10^−3^
	Boceprevir + PR	1	no	65.4 (1179)	43.5 (85)	5.0×10^−5^
		1	yes	44.3 (140)	0 (10)	6.0×10^−3^
	Simeprevir + PR	1	no	83.3 (684)	49.6 (252)	1.2×10^−25^
		1	yes	60.4 (48)	24.6 (61)	1.5×10^−4^
		1	nd	91.3 (150)	52 (50)	5.5×10^−10^
		4	nd	82.9 (35)	40 (40)	1.6×10^−4^
IFN-free regimen	Sofosbuvir + ribavirin	1	no	84 (25)	10 (10)	4.3×10^−5^
	Simeprevir + sofosbuvir	1	no	94.7 (226)	94.1 (17)	9.2×10^−1^
		1	yes	86.4 (176)	100 (4)	4.3×10^−1^
	Ledipasvir + sofosbuvir	1	yes	92.1 (573)	70 (10)	1.2×10^−2^
		1	nd	99.1 (214)	98.1 (49)	2.2×10^−3^
	Ledipasvir + sofosbuvir + ribavirin	1	nd	97.2 (217)	95.7 (46)	5.7×10^−1^
	Ombitasvir + paritaprevir/ritonavir + dasabuvir	1	no	95.7 (983)	100 (32)	2.3×10^−1^
	Ombitasvir + paritaprevir/ritonavir + dasabuvir + ribavirin	1	no	96.4 (1892)	96.3 (188)	9.3×10^−1^
		1	yes	96.7 (418)	86.7 (75)	2.2×10^−4^
	Grazoprevir + elbasvir	1	yes	96.4 (137)	92.9 (14)	5.2×10^−1^
		1	no	92.9 (85)	89.5 (19)	6.1×10^−1^
		1, 4 and 6	nd	94.4 (517)	91.8 (49)	4.7×10^−1^
	Grazoprevir + elbasvir + ribavirin	1	yes	96.9 (32)	90.9 (11)	4.2×10^−1^
		1	no	97.7 (44)	100 (21)	4.9×10^−1^
	Paritaprevir/ritonavir + dasabuvir + ribavirin	1	no	94.7 (19)	47.1 (17)	1.4×10^−3^
	Ombitasvir + paritaprevir/ritonavir	1b	nd	95.2 (42)	89.7 (58)	3.1×10^−1^
	Daclatasvir + simeprevir	1b	nd	84.9 (53)	95 (20)	2.4×10^−1^
	Daclatasvir + simeprevir + ribavirin	1b	nd	74.5 (51)	69.6 (23)	6.6×10^−1^
	Daclatasvir + asunaprevir	1	no	89.5 (171)	79.6 (142)	1.5×10^−2^
		1	yes	90.6 (32)	87.3 (63)	6.3×10^−1^
	Sofosbuvir + radalbuvir + ribavirin	1	no	92 (25)	100 (10)	3.6×10^−1^
	Daclatasvir + asunaprevir + beclabuvir	1	no	92.0 (312)	88.0 (25)	4.9×10^−1^
	Daclatasvir + asunaprevir + beclabuvir ± ribavirin	1	yes	95.5 (112)	97.1 (35)	6.8×10^−1^

^$^nd–not distinguished/determined

Here, we hypothesized that the responsiveness of individuals to IFN and DAAs are causally linked. DAAs are susceptible to viral mutation-driven development of drug resistance [8, 9]. Resistance-associated amino acid variants (RAVs) have been identified that possess high level resistance (>1000-fold increase in EC_50_) to DAAs [9]. Given the rapid turnover of HCV *in vivo* [10] and its high mutation rate [11], RAVs are likely to pre-exist in chronically infected individuals [12] and/or arise during treatment [13], potentially lowering the efficacy of DAAs. Indeed, in retrospective analyses, RAVs were detected more frequently in individuals who failed DAA treatment than in those who achieved SVR [1]. With the combination of ledipasvir and sofosbuvir, for example, 16% of all the patients treated had detectable RAVs at baseline, whereas of those who suffered virological failure, 43% had detectable RAVs at baseline [14]. Although systematic resistance testing is not recommended, current treatment guidelines suggest resistance testing, where such testing is readily accessible and reliable, in the NS5A region to decide appropriate treatment regimens [3]. IFN, a cytokine produced in response to viral infections, triggers the expression of several hundred IFN-stimulated genes (ISGs) in infected cells, creating an antiviral state that restricts viral replication [4, 15]. Higher responsiveness to IFN may thus restrict viral replication to a greater extent, exerting better control on RAVs and leading to improved outcomes of DAA-based treatments. This causal relationship may underlie the positive correlation between responsiveness to PR and DAAs observed in clinical trials. We tested this hypothesis using mathematical modelling and analysis of clinical data.

Mathematical models of HCV kinetics have played a crucial role in our understanding of HCV infection and guided treatments [16]. A model to test the hypothesis above had to address the following questions. 1) What is the origin of the differential responsiveness of HCV-infected individuals to IFN? 2) How can the IFN-responsiveness of an individual be quantified? 3) Given the IFN-responsiveness of an individual, how does the individual respond to DAAs, assuming the hypothesized causal link above? 4) How is IFN-responsiveness distributed across individuals in a population? 5) Does this latter distribution, coupled with the predicted responses of individuals to treatments, explain the differences in SVR rates between treatment-naive and treatment-experienced populations observed across DAAs and across clinical trials?

Existing models [5, 10, 12, 17–27] have addressed some but not all of these questions. For instance, IFN-responsiveness has been shown recently to be an emergent property of the IFN signaling network in HCV infected cells [17]. Due to the competing interactions between ISGs and HCV [28–33], the network exhibits bistability, with one steady state responsive and the other refractory to IFN. The proportion of cells in an individual that preferentially assume the responsive state determines the IFN-responsiveness of the individual [17]. Although variations in ISG protein copy numbers and other factors across cells [25, 34] and across individuals [35, 36] and effects such as those attributed to the polymorphisms in the IL28B gene locus [37] that collectively result in different levels of IFN-responsiveness in different individuals have been identified, how IFN-responsiveness is distributed across individuals remains unknown. Inspired by the success of models in describing HIV drug resistance [38], similar models of HCV kinetics incorporating mutations and their fitness effects have been developed to estimate the pre-existing frequencies of RAVs in chronically HCV infected individuals and to predict their growth during treatment with DAAs [12, 16, 18–22]. The latter models, however, do not treat IFN-responsiveness as a factor influencing the pre-existence and growth of RAVs and hence treatment outcomes. Finally, no models, barring those invoking empirical correlations [20, 39, 40], have described SVR rates elicited by different DAA-based treatment regimens.

Constructing a mathematical model to test the proposed causal relationship thus faced two broad challenges. First, phenomena spanning multiple length and time scales–from the cellular to the population level–had to be integrated into a single mathematical framework. Second, several missing pieces in the puzzle, not considered by existing models, had to be identified. We developed a model that overcame both these challenges. Model predictions captured the vast body of clinical data of the differential response of patients to DAA-based treatments quantitatively, making a strong case for the proposed causal relationship. The model additionally explained several longstanding but poorly understood clinical observations, presenting a far more comprehensive understanding of HCV infection and treatment response than earlier. Finally, using the model, we suggest new strategies, exploiting the causal relationship, to improve DAA-based treatments.

## Results

### Correlation between responsiveness to PR and DAAs

To establish the correlation between the responsiveness of chronically HCV infected individuals to PR and DAAs, we collated data from all (over 50) clinical trials that reported SVR rates achieved with DAA-based treatments in treatment-naïve individuals, *SVR*_*naive*_, and in previous null responders to PR, *SVR*_*null*_ (Methods). The data are grouped according to treatment regimen and summarized in Table 1. Individual datasets are listed in S1 Table. We found that *SVR*_*naive*_>*SVR*_*null*_ with P≈10^−59^ overall (using the χ^2^-test). The difference was starker when the analysis was restricted to treatments that included PR (P≈10^−65^), but, importantly, was highly significant when interferon-free regimens alone were considered (P = 0.007). The difference remained when only individuals with (S2 Table; P≈10^−5^) or without (S3 Table; P≈10^−29^) liver cirrhosis were considered or when the analysis was restricted to studies that did not factor liver cirrhosis (S4 Table; P≈10^−30^). The difference was clearer for treatments that elicited <100% SVR than for more recent, stronger treatments that elicited ~100% SVR regardless of treatment experience. Nonetheless, the clinical evidence of a positive correlation between responsiveness to PR and DAAs was overwhelming and suggested a causal relationship between the two. We proposed a mechanistic hypothesis underlying this relationship, where greater IFN-responsiveness exerted better control on RAVs and improved DAA treatment outcomes (see above), and constructed a mathematical model to test it.

### Mathematical model

**Fig 1:**
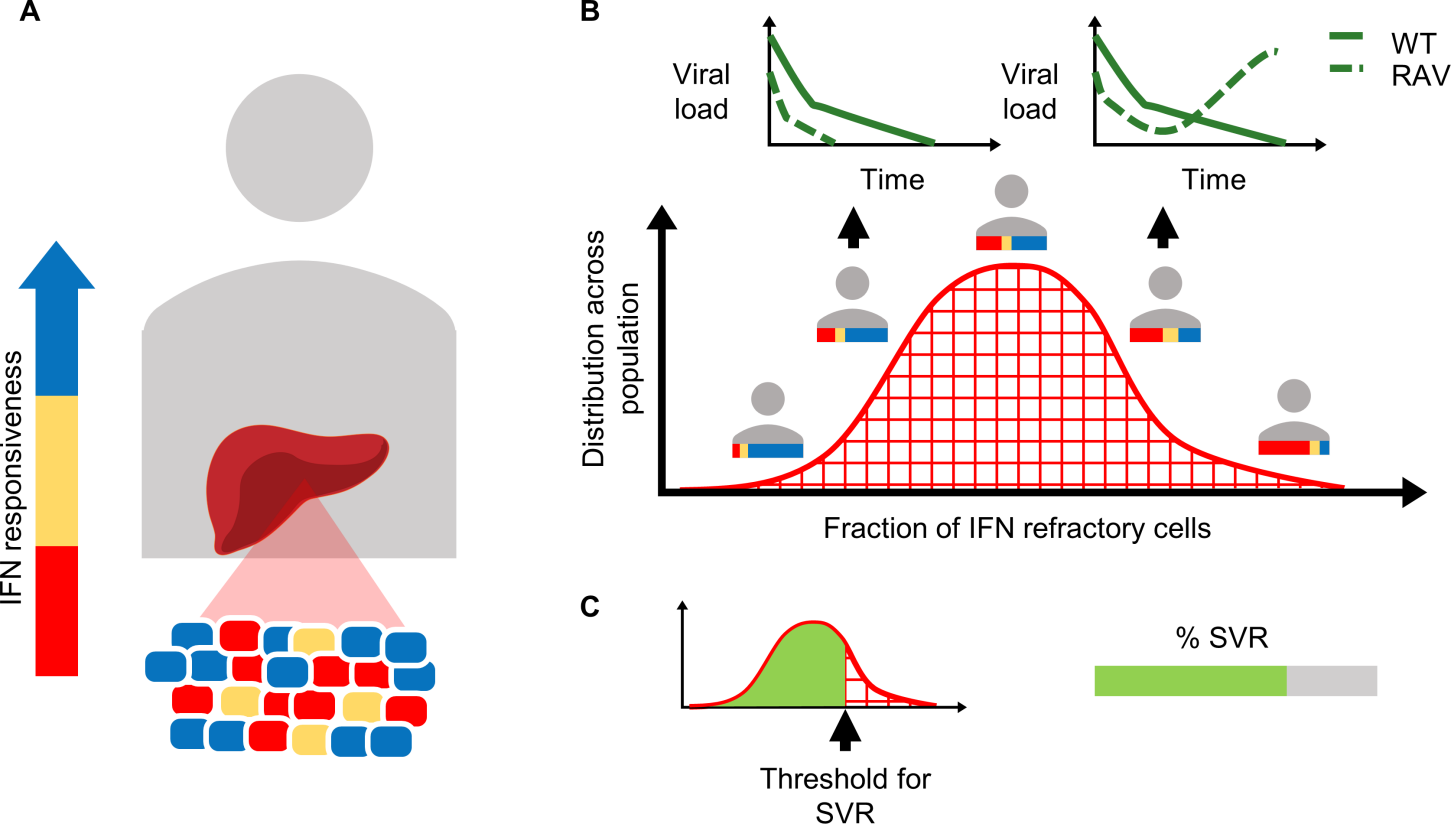
Schematic of the model. **(A)** Hepatocytes in an HCV infected individual display a range of phenotypic responses to IFN, from refractory (red) to responsive (blue). **(B)** Different individuals carry different fractions of hepatocytes displaying these distinct responses, yielding a distribution of IFN-responsiveness in an HCV infected population. Individuals with a small proportion of IFN-refractory hepatocytes respond to treatment (left inset), whereas those with a large proportion see virologic breakthrough due to the growth of drug resistant (RAV) and/or wild-type (WT) strains (right inset). **(C)** The threshold or admissible proportion of IFN-refractory hepatocytes depends on the drugs and treatment protocol employed and defines the SVR rate the treatment elicits in a population.

We present an overview of the model here (Fig 1). A detailed description of the various components of our model and how we integrated them into a single framework is in Methods.

To describe the response of an individual to PR, we employed the formalism we developed previously, where cells were divided into distinct IFN-responsive and IFN-refractory phenotypes based on the properties of the IFN-signaling network in HCV-infected cells [17]. At the cellular level, interferon triggers the expression of several hundred interferon-stimulated genes (ISGs) that collectively create an antiviral state in cells [15]. HCV suppresses the interferon response via multiple mechanisms [4, 41], the prominent one involving a block in ISG translation it induces via dimerization and autophosphorylation of protein kinase R [30, 42, 43]. We constructed a comprehensive model of the IFN signaling network in the presence of HCV, accounting for the competing effects above which amounted to a double negative feedback, and found that the network exhibited bistability [17]. In one steady state, HCV overcame the IFN response and established lasting infection. In the other, IFN cleared HCV. Intrinsic variations of the factors defining the IFN signaling network, which defined the strength of the IFN response relative to the strength of its subversion by HCV, resulted in individual cells admitting either one or both the states. Cells that admitted the first steady state alone were refractory to IFN. Cells that admitted the latter alone were responsive to IFN. Cells that admitted both were bistable and the state they eventually realized depended on whether they were exposed earlier to HCV or IFN.

Based on the description above, we divided cells in an individual into three distinct IFN-response phenotypes, IFN-refractory, bistable, and IFN-responsive. With this classification, we constructed a model of viral kinetics that described viral load changes in individuals following the onset of PR treatment. HCV thrived in the IFN-refractory compartment despite exposure to PR. The relative proportion of cells that were of the IFN-refractory phenotype thus quantified the IFN-responsiveness of individuals. By increasing this proportion, which decreased IFN-responsiveness, the formalism was shown to quantitatively capture the observed patterns of viral load decline, from rapid response to null response, in patients undergoing PR treatment [17].

To describe the pre-existence and growth of RAVs under DAA-based treatment, we built on previous models of viral kinetics and evolution [12, 16, 18–22], which have provided good fits to patient data of wild-type and RAV population dynamics [12, 16]. The models allowed mutations, which occurred during viral replication, at specific loci to confer resistance to DAAs. The mutations, however, came with fitness costs. The models could thus predict the pre-existing frequencies of RAVs and their growth rates during treatment with DAAs. We combined the models above by integrating the distinct cellular phenotypes with viral kinetics and evolution to arrive at a description of the response of an individual to DAA-based treatments and the influence IFN has on the response. The poorer the IFN-responsiveness of an individual, the greater the level of ongoing replication during treatment, and hence the higher the likelihood of the development of resistance to DAAs. The combined model thus yielded threshold levels of IFN-responsiveness required for treatments to succeed.

We next developed independent descriptions of the distribution of IFN-responsiveness in populations. We showed that the pre-treatment set-point viral load was directly related to the IFN-responsiveness of an individual, allowing us to quantify the distribution of IFN-responsiveness using measurements of viral load in populations. The fraction of individuals with IFN-responsiveness above the threshold IFN-responsiveness predicted above for any treatment yielded the SVR rates elicited by that treatment. In particular, the threshold for a null response to PR yielded the percentage of null responders and hence, truncating the distribution above to the latter threshold, the distribution of IFN-responsiveness in null responders to PR. Linking the distribution of IFN-responsiveness in populations to the individual-level models of viral kinetics and treatment response thus allowed estimation of SVR rates across different populations, particularly treatment naïve and previous null responders to PR, elicited by different treatment regimens.

### Pre-treatment RAV frequencies

**Fig 2:**
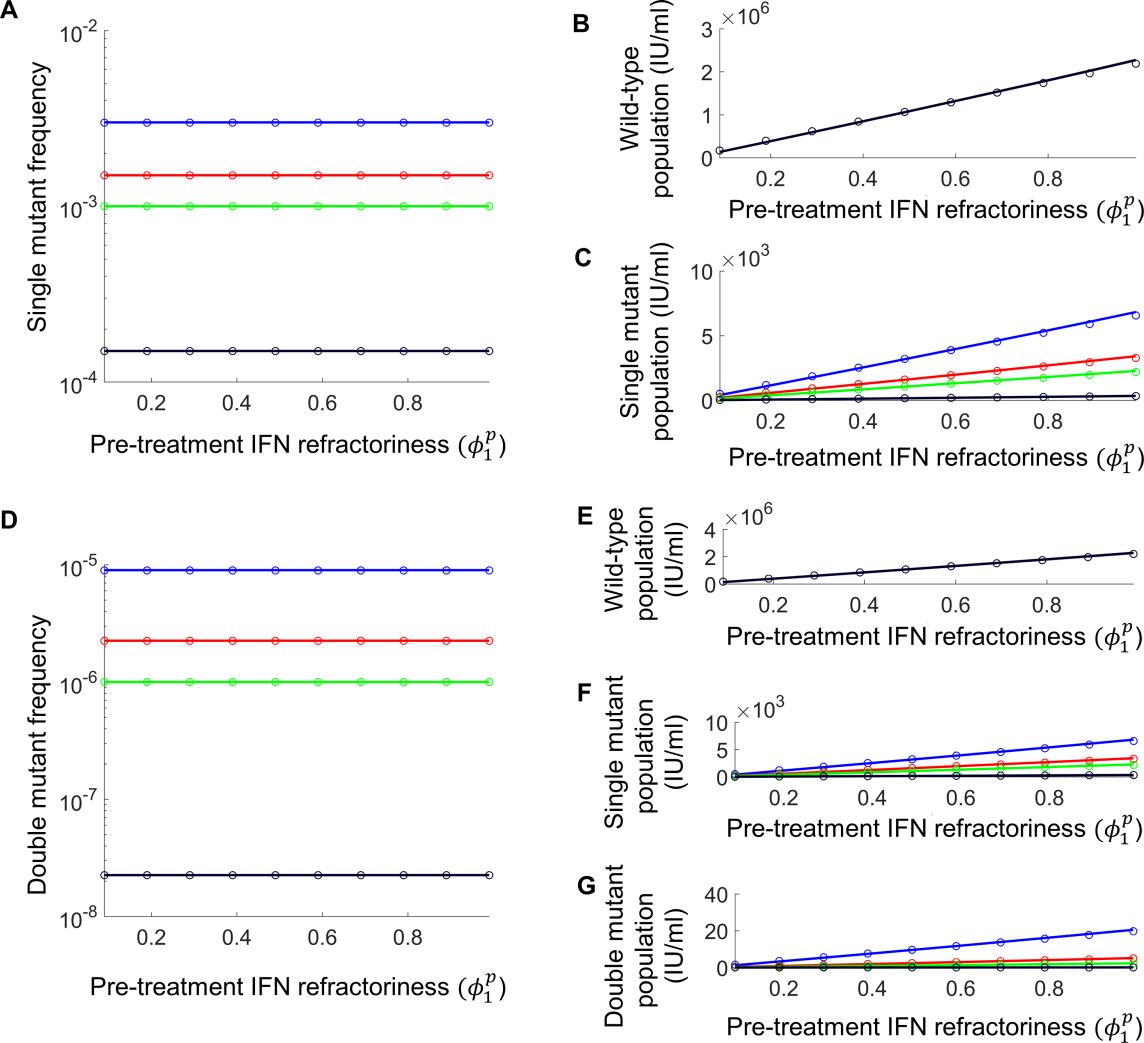
Pre-treatment frequencies and populations of virions. Model predictions (lines) and analytical approximations (symbols) (S1–S3 Texts) of the mutant frequencies (left) and viral populations (right) in the pre-treatment steady state as a function of the level of IFN-responsiveness, $\phi_{1}^{p}$, for different combinations of the mutation rate, *μ*, and the relative fitness of the RAV, *γ*: (*μ*,*γ*) = (3×10^−4^,0.9) (blue), (3×10^−4^,0.8) (red), (3×10^−4^,0.7) (green) and (3×10^−5^,0.8) (black). Here, *γ* = *p*_1_/*p*_0_, the ratio of the viral production rates, or equivalently the replicative abilities, of the mutant and wild-type strains; without loss of generality, the RAV was assumed not to compromise viral infectivity (see S1–S3 Texts). Single mutant frequencies (**A)** and the populations of wild-type **(B)** and single mutant **(C)** virions when the genetic barrier is 1. Double mutant frequencies **(D)** and the populations of wild-type **(E)**, single mutant **(F)**, and double mutant **(G)** virions when the genetic barrier is 2. In the latter case, the two single mutants have the same relative fitness, *γ*, and the double mutant, *γ*^2^. In (B) and (E), the different lines and symbols are indistinguishable. Parameter values employed are in S5 Table. The parameters to which these predictions are sensitive are as expected from the analytical approximations (S1 Fig).

We applied the model first to examine whether greater IFN-responsiveness lowered the pre-existence of RAVs in infected individuals (Fig 2). We quantified the IFN-responsiveness of an individual by the fraction of target cells produced in the individual that exhibited the IFN-refractory phenotype [17], denoted $\phi_{1}^{p}$. The smaller the value of $\phi_{1}^{p}$, the more IFN-responsive was the individual. Using our model, we estimated the steady state pre-treatment populations of wild-type and RAV-carrying virions, *V*_0_ and *V*_1_, respectively, and the frequency, *ρ*_1_ = *V*_1_/(*V*_0_+*V*_1_), of RAVs, as functions of $\phi_{1}^{p}$ (Eqs (1)–(4), Methods). A single point mutation was assumed first to confer resistance to the DAA. Mutation occurred at the rate *μ* and allowed the production of *V*_1_ from cells infected with *V*_0_. The mutations came with a fitness cost to the virus, determined by lower values of viral infectivity and/or productivity, *γ*, relative to the wild-type. We found that *ρ*_1_ was independent of $\phi_{1}^{p}$ (Fig 2A). As $\phi_{1}^{p}$ increased, fewer cells were IFN-responsive, which resulted in an increase in *V*_1_ (Fig 2C). However, of the virions produced (Fig 2B and 2C), a constant fraction, determined by the mutation rate, *μ*, and the relative fitness of the RAV, *γ*, carried the RAV, leaving *ρ*_1_ unaffected. Further, both *V*_1_ and *ρ*_1_ but not *V*_0_ increased significantly with *μ* and *γ* (Fig 2A–2C). We derived analytical approximations (S1 Text) that quantitatively explained these variations (Fig 2A–2C). The results were readily extended to multiple loci (S2 and S3 Texts; Fig 2D–2G).

Thus, greater IFN-responsiveness did not significantly alter the pre-treatment frequencies of RAVs, although it did lead to lower pre-treatment viral loads and populations of RAVs, indicating greater control over ongoing viral replication. This greater control could influence the growth of RAVs during treatment with DAAs, which we examined next.

### Growth of RAVs during IFN-free treatment

**Fig 3:**
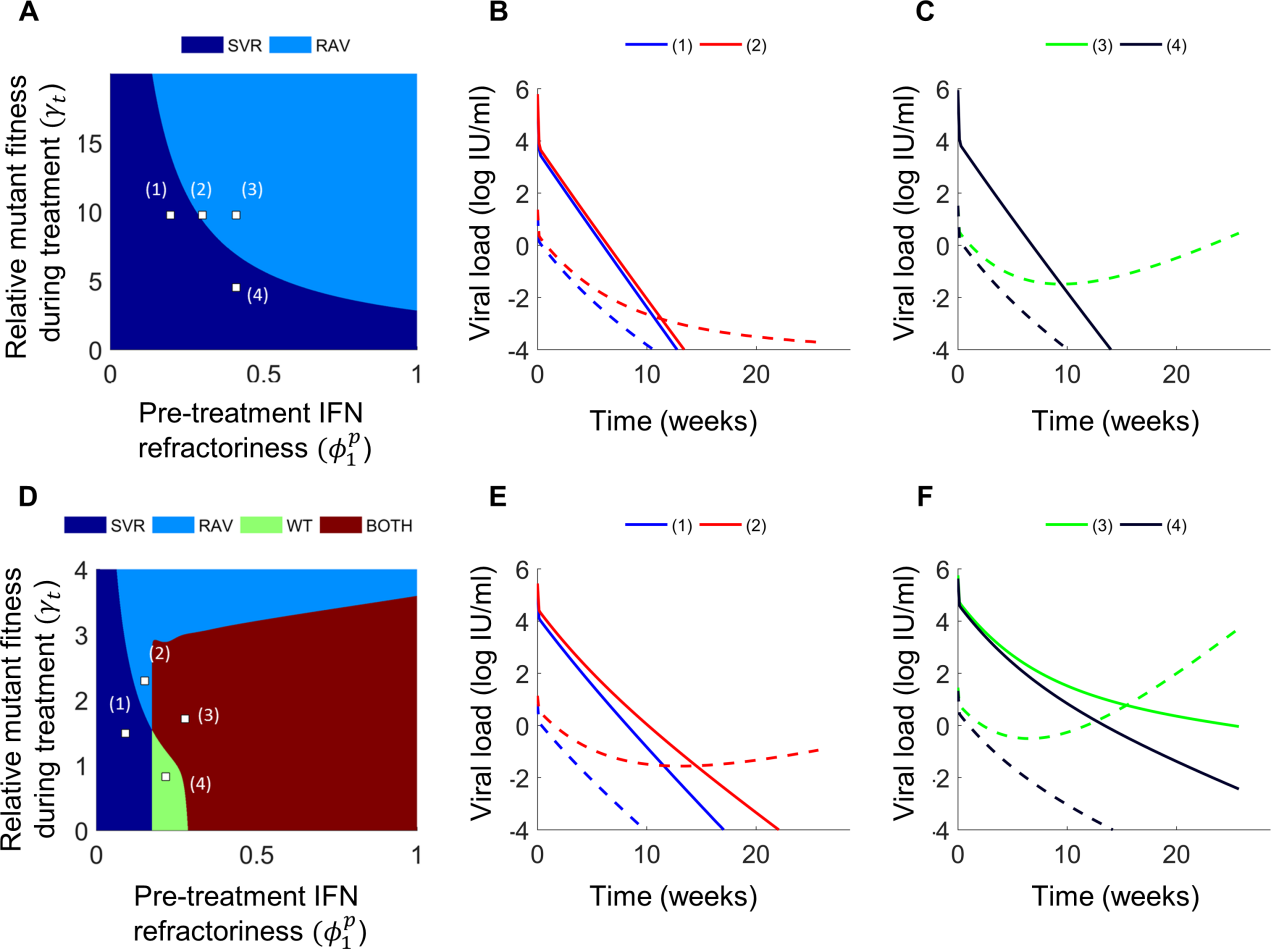
Response to IFN-free DAA treatment. **(A)** Phase diagram indicating regimes of the level of IFN-responsiveness, $\phi_{1}^{p}$, and the relative fitness of the RAV during treatment, *γ*_*t*_, that lead to SVR (dark blue) or treatment failure due to virological breakthrough by the RAV (light blue). **(B,C)** Dynamics of wild-type (solid) and RAV (dashed) viral populations following treatment initiation for different parameter combinations numbered in (A). Here, the efficacy of treatment against the wild-type is assumed to be high: $\varepsilon_{DAA}^{0}=0.99$. Also, *γ* = 0.2. **(D)** Phase diagram with lower efficacy, $\varepsilon_{DAA}^{0}=0.9$, showing regions leading to SVR (dark blue) or treatment failure due to the RAV (light blue), wild-type (green), or both (brown). **(E,F)** Dynamics of wild-type (solid) and RAV (dashed) viral populations for the points numbered in (D). Here, *γ* = 0.4. Other parameter values employed are in S5 Table. Phase diagrams for other values of $\varepsilon_{DAA}^{0}$ are in S2 Fig.

We predicted the viral load decline under DAA treatment for a range of treatment efficacies against the wild-type, $0\leq \varepsilon_{DAA}^{0}\leq 1$, and the RAV, $0\leq \varepsilon_{DAA}^{1}\leq \varepsilon_{DAA}^{0}$, and for different fitness penalties, *γ*, associated with the RAV (Eqs (1)–(4), Methods). We defined the effective relative fitness of the RAV during treatment as $\gamma_{t}=\gamma (1-\varepsilon_{DAA}^{1})/(1-\varepsilon_{DAA}^{0})$, combining the intrinsic fitness disadvantage of the RAV and its advantage in the presence of the drug. We defined $\phi_{1}^{t}$ as the IFN-responsiveness during treatment. $\phi_{1}^{t}$ depended on the total IFN exposure, the sum of endogenous and exogenous levels [17]. For IFN-free treatments, where exogenous IFN is absent, we let $\phi_{1}^{t}=\phi_{1}^{p}$ (Methods). We found that the response to treatment was determined predominantly by $\varepsilon_{DAA}^{0}$, $\phi_{1}^{p}$ and *γ*_*t*_ (Fig 3). With high $\varepsilon_{DAA}^{0}(∼0.99)$, the wild-type could be cleared by the DAA regardless of $\phi_{1}^{p}$. Then, for any *γ*_*t*_, the decline of the RAVs was faster for lower $\phi_{1}^{p}$. Below a critical value of $\phi_{1}^{p}$, SVR was achieved, whereas above this critical value, virological breakthrough occurred (Fig 3A and 3C). Similarly, for a fixed $\phi_{1}^{p}$, RAVs declined faster for lower *γ*_*t*_ (Fig 3A and 3B). A locus of points on a $\phi_{1}^{p}-\gamma_{t}$ plot delineated the region where SVR occurred from that where virological failure resulted due to drug resistance (Fig 3A). For lower $\phi_{1}^{p}$, breakthrough occurred at higher *γ*_*t*_, indicating that higher degrees of resistance were necessary for virological breakthrough with higher IFN-responsiveness.

With lower $\varepsilon_{DAA}^{0}$, the DAA was not potent enough to suppress the wild-type at all $\phi_{1}^{p}$. With $\phi_{1}^{p}$ large and *γ*_*t*_ small (<1), the wild-type drove failure (Fig 3D and 3F). The value of $\phi_{1}^{p}$ above which failure occurred decreased as $\varepsilon_{DAA}^{0}$ decreased, indicating that failure occurred even with higher IFN-responsiveness as the DAA efficacy dropped (Fig 3A and 3D). Conversely, poorer IFN-responsiveness placed more stringent demands on the DAA. As *γ*_*t*_ increased, both the wild-type and RAV co-existed during treatment failure and when *γ*_*t*_ rose above ~1, the RAV outcompeted the wild-type and drove treatment failure (Fig 3D and 3E).

### Growth of RAVs during PR+DAA treatment

**Fig 4:**
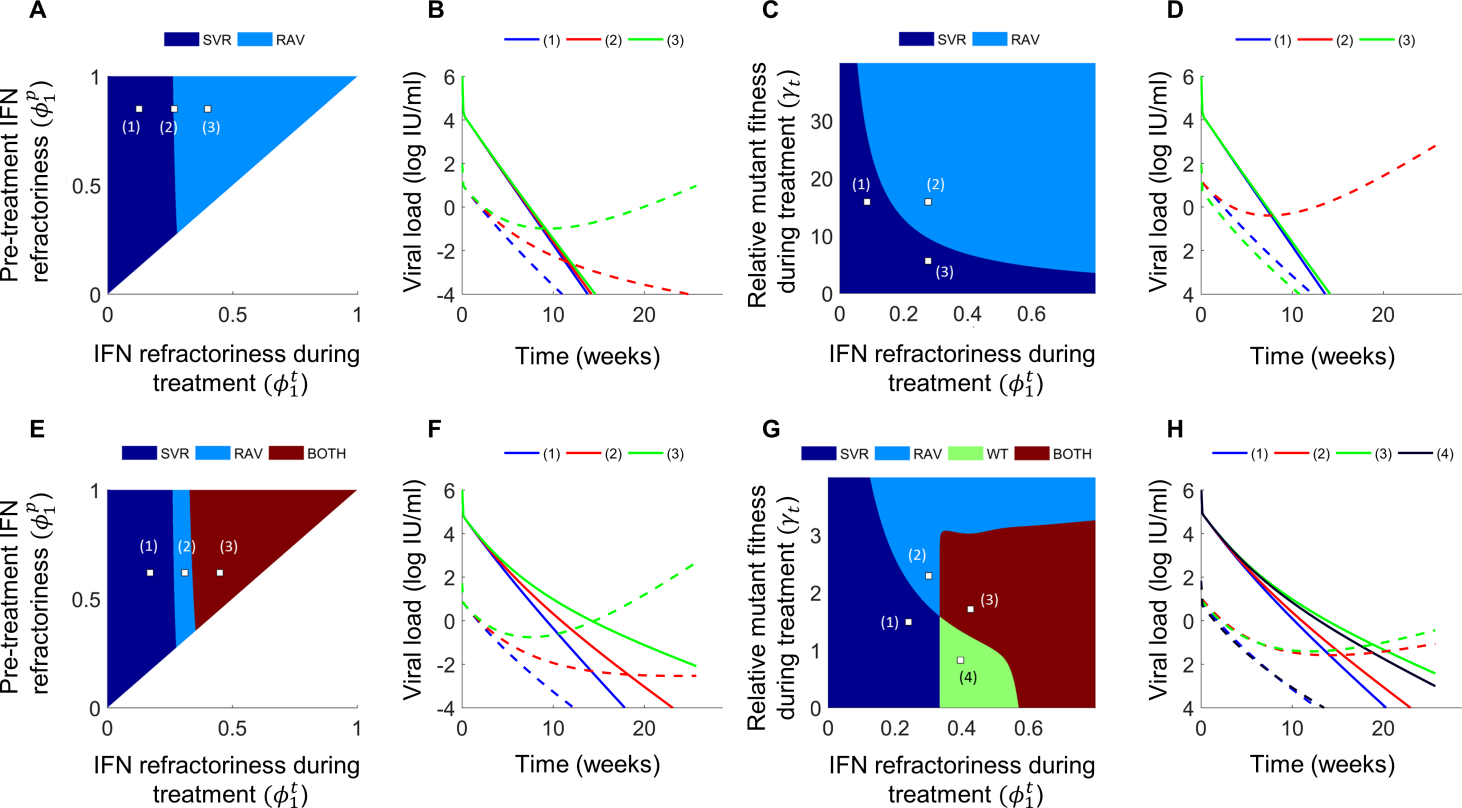
Response to PR+DAA treatment. **(A)** Phase diagram indicating regimes of IFN-responsiveness pre- and during treatment, $\phi_{1}^{p}$ and $\phi_{1}^{t}$, leading to SVR (dark blue) and treatment failure due to virological breakthrough by the RAV (light blue) for a fixed relative fitness of the RAV during treatment, *γ*_*t*_. **(B)** Dynamics of wild-type (solid) and RAV (dashed) viral populations following treatment initiation for parameter combinations numbered in (A). **(C)** Phase diagram on a $\phi_{1}^{t}-\gamma_{t}$ plot for fixed $\phi_{1}^{p}$. **(D)** Dynamics for the points numbered in (C). In (A)-(D), the DAA efficacy against the wild-type, $\varepsilon_{DAA}^{0}=0.99$. Also, *γ* = 0.4. **(E)-(H)** Corresponding predictions with $\varepsilon_{DAA}^{0}=0.95$. In (E) and (G), treatment failure occurred due to the RAV (light blue), wild-type (green), or both (brown). Here, *γ* = 0.2. Other parameter values employed are in S5 Table. Phase diagrams for other values of $\varepsilon_{DAA}^{0}$ are in S3 Fig.

With exogenous IFN present, $\phi_{1}^{t}<\phi_{1}^{p}$. We therefore let $0\leq \phi_{1}^{p}\leq 1$ and $0\leq \phi_{1}^{t}\leq \phi_{1}^{p}$ (Fig 4). For fixed $\varepsilon_{DAA}^{0}$, *γ*_*t*_ and $\phi_{1}^{p}$, RAVs declined faster for lower $\phi_{1}^{t}$ (Fig 4B). Again, a threshold $\phi_{1}^{t}$ existed below which SVR was achieved and above which RAVs drove virological breakthrough when $\varepsilon_{DAA}^{0}$ was high (Fig 4A). This threshold was weakly sensitive to $\phi_{1}^{p}$ because pre-treatment variations were rapidly subsumed post treatment initiation by the dynamics dictated by $\phi_{1}^{t}$ (Fig 4B). The threshold, however, was sensitive to *γ*_*t*_. As *γ*_*t*_ increased, the threshold dropped, indicating that treatment failure occurred even with higher IFN-responsiveness as the RAVs became more fit (Fig 4C and 4D). Similarly, as $\varepsilon_{DAA}^{0}$ decreased, failure occurred at lower $\phi_{1}^{t}$, indicating again that poorer IFN-responsiveness contributed to the failure of DAAs (Fig 4E and 4F). Further, as *γ*_*t*_ increased from <<1 to >>1, failure occurred first due to the wild-type, then the combination of wild-type and RAVs, and finally due to the RAVs alone (Fig 4G and 4H).

Thus, with DAA-based treatment, with or without PR, IFN-responsiveness controlled the growth of RAVs and contributed to treatment success. We examined next the implications of these findings at the population level. For this, we first estimated the distribution of IFN-responsiveness across individuals.

### Distribution of IFN-responsiveness in patient populations

**Fig 5:**
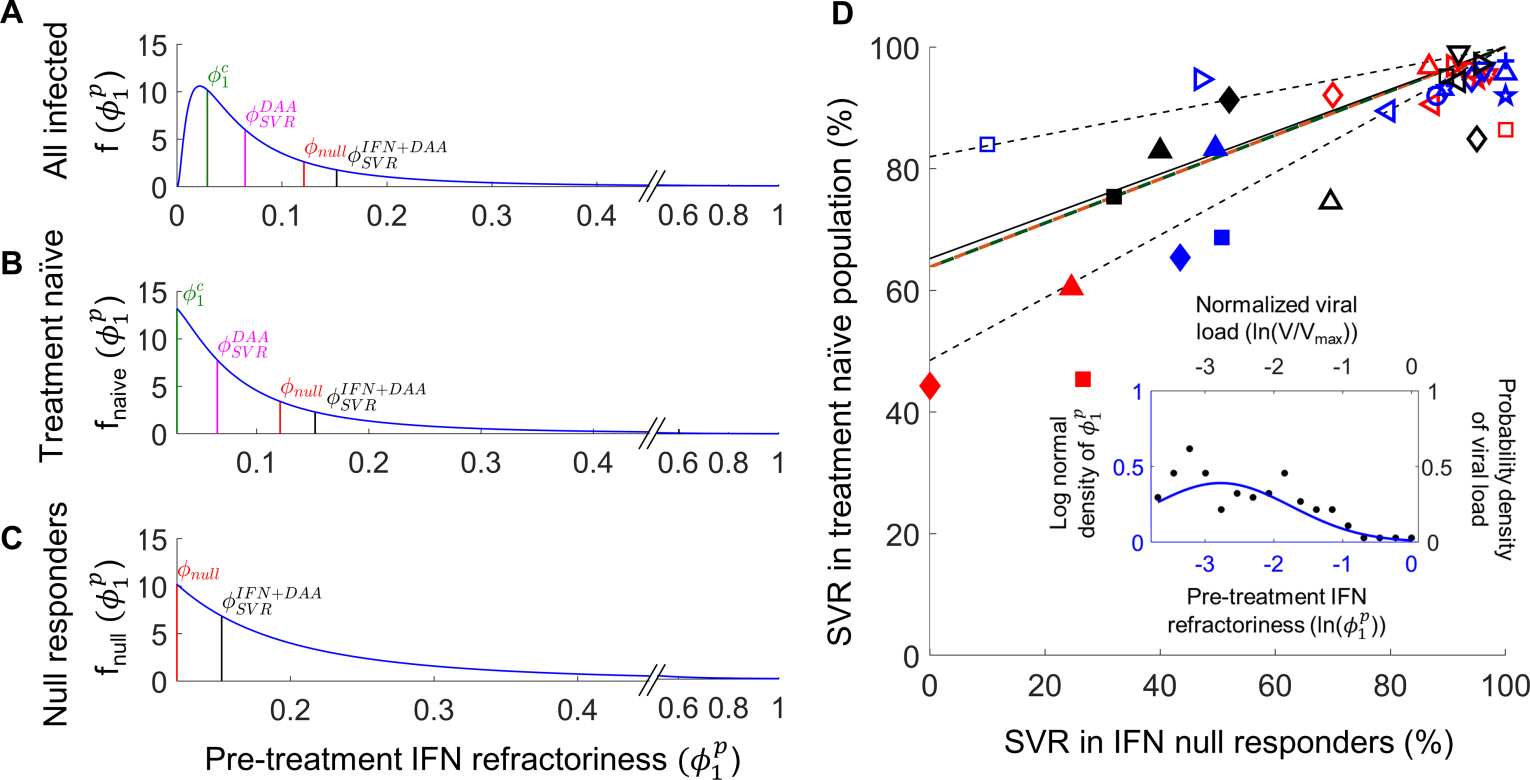
Distribution of IFN-responsiveness across patients and SVR rates to DAA-based treatments. **(A)** The log-normal probability density of the level of IFN-responsiveness, $\phi_{1}^{p}$, across individuals. The values of $\phi_{1}^{p}$ below which we observe spontaneous clearance, $\phi_{1}^{c}$, and below which the DAA would elicit SVR either alone, $\phi_{SVR}^{DAA}$, or with PR, $\phi_{SVR}^{PR+DAA}$, are indicated. $\phi_{1}^{p}$ above which PR would elicit a null response, *ϕ*_*null*_, is also indicated. The resulting probability density of $\phi_{1}^{p}$ in **(B)** treatment-naïve patients and **(C)** prior null responders to PR. **(D)** Comparisons of our model predictions (lines) of SVR rates in treatment-naive versus prior null responders to PR with corresponding observations from clinical trials involving IFN-free DAA regimens (open symbols) or PR+DAA combinations (filled symbols). Studies either distinguished individuals with (red) and without (blue) liver cirrhosis or not (black). The clinical data is collated in Tables 1 and S1. Model predictions (Eqs (1)–(13), Methods) with (orange) and without (green) PR overlap. The best-fit (black solid) along with 95% CI (black dashed) of Eq (18) to the data where cirrhosis is not distinguished (collated in S4 Table) is shown. *Inset*: The log-normal probability density of $\phi_{1}^{p}$ in treatment-naïve individuals (line) fit to data of baseline viral loads from patients (symbols). The best-fit parameters were *ν* = −2.775 and *σ* = 1.027 (Methods).

We recognized that $\phi_{1}^{p}$ was linked directly to the chronic set-point viral load in our model (Methods; Eq. (S1.10)). The set-point viral load has been found to be log-normally distributed in chronically-infected individuals [44]. We therefore let $\phi_{1}^{p}$ also be log-normally distributed (Eq (5); Fig 5A). Chronic infection was only possible in our model when $\phi_{1}^{p}$ was larger than a threshold, $\phi_{1}^{c}$. When $\phi_{1}^{p}\leq \phi_{1}^{c}$, the set-point viral load was zero, marking spontaneous clearance of infection. Using representative model parameter values, we solved our model (Eqs (1)–(4), Methods) using different values of $\phi_{1}^{p}$ and identified $\phi_{1}^{c}$ as the maximum value of $\phi_{1}^{p}$ for which the set-point viral load vanished. We thus obtained $\phi_{1}^{c}=0.029$. We next fit a truncated log-normal distribution for $\phi_{1}^{p}>\phi_{1}^{c}$ (Eq (6), Fig 5B) to patient data of the distribution of set-point viral load and identified parameter values defining the log-normal distribution of $\phi_{1}^{p}$ in a treatment-naïve population (Fig 5D, inset).

With the resulting distribution, we estimated the percentage of individuals with $\phi_{1}^{p}\leq \phi_{1}^{c}$ (Eq (8)), *i*.*e*., the fraction of infected individuals who spontaneously clear the infection, and found it to be ~21%, close to the mean of ~26% obtained from 31 longitudinal studies [45].

We next considered null responders to PR, defined by $\phi_{1}^{p}>\phi_{null}$. To estimate *ϕ*_*null*_, we employed clinical data of telaprevir-based treatments. Using parameter values similar to previous estimates [12, 46], $\varepsilon_{DAA}^{0}\approx 0.99$, $\varepsilon_{DAA}^{1}\approx 0.03$, and *γ* = 0.4, we applied our model (Eqs (1)–(4)) and estimated $\phi_{SVR}^{DAA}=0.065$ as the value of $\phi_{1}^{p}$ below which telaprevir monotherapy would elicit SVR (Methods). (Note that telaprevir monotherapy can induce SVR [47].) We next estimated $\phi_{SVR}^{PR+DAA}$, the value of $\phi_{1}^{p}$ below which PR+telaprevir triple therapy would yield SVR, by comparing model predictions of SVR rates in treatment-naïve patients (Eq (9)) with corresponding clinical data [48], $SVR_{naive}^{PR+DAA}=75.4\pm 2.4\%$. Given the distribution of $\phi_{1}^{p}$, the value of $\phi_{1}^{p}$ below which a defined percentage of the population lies can be calculated. Thus, the value of $\phi_{1}^{p}$ below which $SVR_{naive}^{PR+DAA}=75.4\pm 2.4\%$ of the population lies yielded $\phi_{SVR}^{PR+DAA}=0.152\pm 0.011$. This implied $\Delta \phi =\phi_{SVR}^{PR+DAA}-\phi_{SVR}^{DAA}=0.087\pm 0.011$ was the increase in IFN-responsiveness due to exogenous IFN administered as part of PR treatment. With this Δ*ϕ*, we could estimate $\phi_{1}^{t}$ for any $\phi_{1}^{p}$, allowing us to use our model (Eqs (1)–(4)) to predict the response to PR-containing regimens. In particular, we could predict the response to PR. Solving our model, we thus identified *ϕ*_*null*_ as the minimum $\phi_{1}^{p}$ that yielded a null response to PR, defined as having occurred when <2 log_10_ decline in viral load resulted from 12 weeks of treatment. We found that *ϕ*_*null*_ = 0.12±0.01 (Eqs (1)–(4), Methods). Truncating the distribution of $\phi_{1}^{p}$ to values of $\phi_{1}^{p}$ above *ϕ*_*null*_ yielded the distribution of $\phi_{1}^{p}$ in null responders to PR (Eq (7), Fig 5C).

With these estimates, we predicted the percentage of null responders to PR in a treatment-naïve population as the fraction of the population with $\phi_{1}^{p}$ above *ϕ*_*null*_ (Eq (11)) and found $NULL_{naive}^{PR}=33\%$, which was in close agreement with corresponding clinical observations of 32% [49]. Further, we predicted the response of null responders to PR+telaprevir triple therapy (Eq (13)) as the fraction of null responders with $\phi_{1}^{p}$ below $\phi_{SVR}^{PR+DAA}$ estimated above, and found $SVR_{null}^{PR+DAA}=26\%$, again in good agreement with the 32% observed experimentally [48].

This quantitative agreement of our model with independent observations gave us confidence in our model and our estimates of the distribution of IFN-responsiveness. In addition, that the same Δ*ϕ* captured the observed influence of PR with and without telaprevir implied that the proposed synergy between IFN and DAAs [17, 50, 51] may be small in vivo. We next predicted the response of different patient subpopulations to DAA-based treatments.

### SVR rates elicited by DAA-based treatments

Using the distributions of IFN-responsiveness identified above, we applied our model (Eqs (1)–(13), Methods) to predict SVR rates elicited by DAA-based treatments. We varied parameters to mimic the entire spectrum of accessible DAA efficacies and relative fitness values of RAVs. We found that *SVR*_*null*_ was consistently lower than *SVR*_*naive*_ (Fig 5D). Further, our predictions were in good agreement with clinical data (Fig 5D; Table 1). The predictions employed some parameters estimated above from telaprevir-based therapy. We derived an analytical expression linking *SVR*_*naive*_ and *SVR*_*null*_ that was independent of the DAA and of whether PR was part of the treatment (Eq (18), Methods): $SVR_{naive}=1-NULL_{naive}^{PR}+NULL_{naive}^{PR}SVR_{null}^{}$. The expression too fit the clinical data well and the fit was close to our predictions above (Fig 5D). The best-fit estimate of $NULL_{naive}^{PR}=40\pm 14\%$ was in agreement with the corresponding clinical estimate [49] of 32%. Further, the latter expression explained more vividly the diminishing difference between *SVR*_*naive*_ and *SVR*_*null*_ as treatment became more potent. It showed that when *SVR*_*naive*_ approached 100%, so did *SVR*_*null*_, in agreement with observations from recent trials where nearly all patients were cured regardless of their treatment experience (Table 1).

This agreement between our predictions, in two ways, and clinical data demonstrating *SVR*_*null*_ ≤ *SVR*_*naive*_ presents strong evidence supporting our hypothesis of the causal relationship between IFN-responsiveness and the success of DAA-based treatments. We examined ways of exploiting this relationship to improve treatments.

### Potential strategies to improve DAA-based treatments

**Fig 6:**
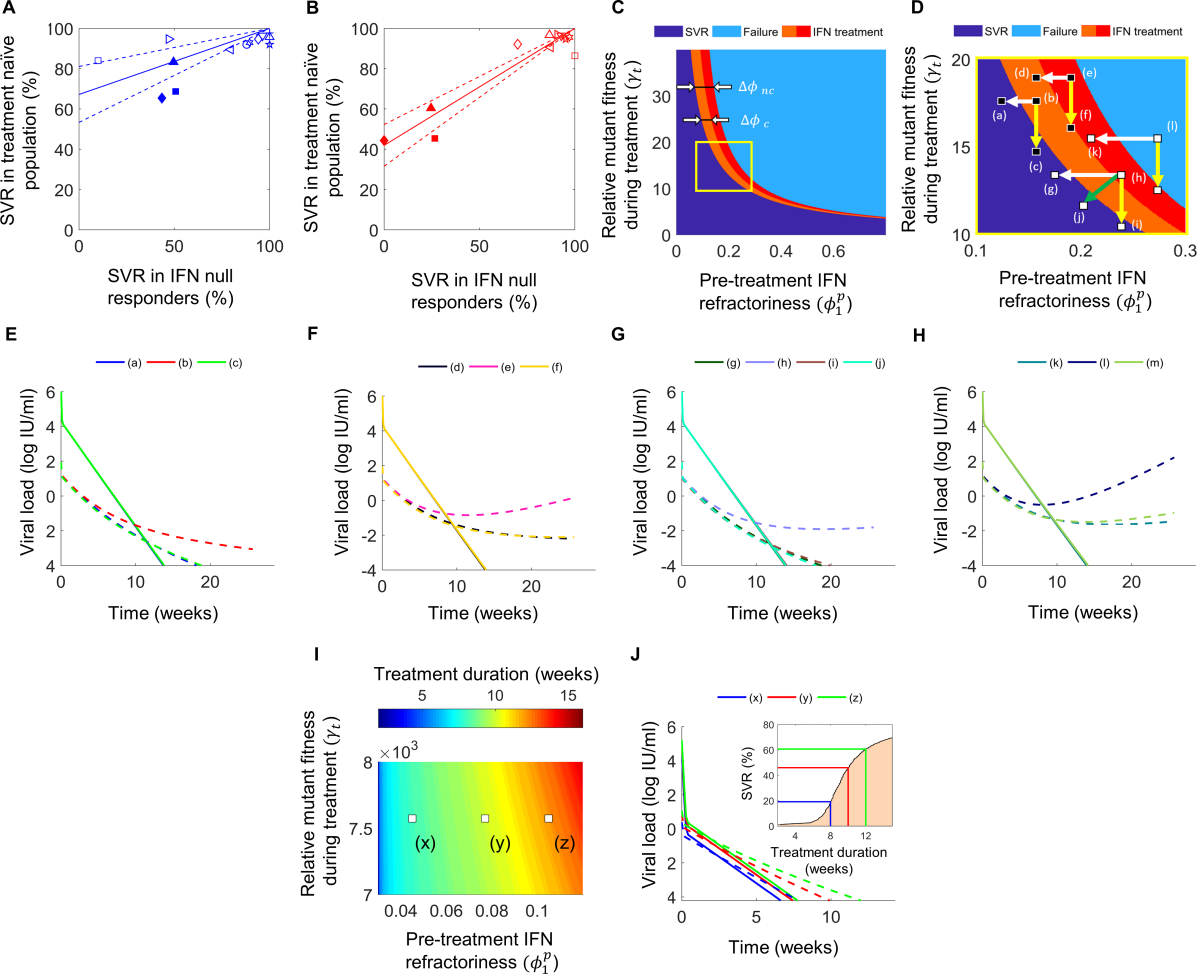
Strategies to overcome DAA failure. The best-fit (solid line) and 95% CIs (dashed lines) of Eq (18) (Methods) to data (symbols) of SVR rates in treatment-naïve versus treatment-experienced patients **(A)** without liver cirrhosis and **(B)** with liver cirrhosis, treated with DAAs with (filled) or without (open) PR. The list of clinical trials from which data has been collated is presented in S2 and S3 Tables, respectively. The fits yielded $NULL_{naive}^{PR}=58\pm 10\%$ and 33±14% in the two subpopulations, respectively, and using which, we estimated the corresponding *ϕ*_*null*_ = 0.07 and 0.12. **(C)** Regions in the phase diagram (see Fig 2A) where addition of PR to the DAA would elicit cure in otherwise failing patients with (orange) or without (orange and red) liver cirrhosis. SVR would be elicited in the dark blue region of the phase diagram. **(D)** The yellow box in (C) zoomed to demonstrate the influence of adding PR to an individual with a cirrhotic (small white arrow) or a non-cirrhotic (large white arrow) liver, adding a new DAA or increasing DAA dosage (yellow arrow), or adding PR and a new DAA (green arrow). **(E-H)** Dynamics of wild-type (solid) and RAV (dashed) viral populations following treatment initiation for the different conditions marked in (D). **(I)** The duration of treatment in weeks required to achieve SVR for a range of values of IFN-responsiveness, $\phi_{1}^{p}$, and the relative fitness of the RAV, *γ*_*t*_. **(J)** Dynamics of wild-type (solid) and RAV (dashed) viral populations following treatment initiation for the different conditions marked in (I), corresponding to daclatasvir treatment (Methods). *Inset*: The percentage of patients predicted to achieve SVR as a function of the duration of treatment with daclatasvir. The percentages corresponding to the conditions marked in (I) are indicated. Thus, 19.2%, 46% and 60.6% SVR rates are expected in 8, 10, and 12 weeks of treatment, respectively.

IFN-responsiveness could be exploited to improve DAA-based treatments in two ways: 1) to prevent failure and 2) to shorten the treatment duration (Fig 6). IFN-free treatment would fail in an individual if $\phi_{1}^{p}$ in the individual were larger than a threshold. Adding PR would lower $\phi_{1}^{p}$ by Δ*ϕ*. Δ*ϕ* is expected to be different for individuals with (denoted Δ*ϕ*_*c*_) and without (denoted Δ*ϕ*_*nc*_) liver cirrhosis. We fit the analytical expression above (Eq (18)) to SVR data on populations with and without liver cirrhosis separately (Fig 6A and 6B) and estimated Δ*ϕ*_*c*_ = 0.046 and Δ*ϕ*_*nc*_ = 0.091 (Methods). PR thus appeared only half as effective in improving IFN-responsiveness in cirrhotic individuals as non-cirrhotic individuals. Adding PR would thus induce SVR if the individual had a cirrhotic (non-cirrhotic) liver and the $\phi_{1}^{p}$ were within Δ*ϕ*_*c*_(Δ*ϕ*_*nc*_) of the threshold (Fig 6C, 6D, 6E and 6G). If $\phi_{1}^{p}$ were farther away from the threshold, adding PR alone would prove inadequate (Fig 6D, 6F and 6H). Increasing the DAA dosage or including additional DAAs to lower the effective fitness of the RAV may then be a way to induce SVR (Fig 6C–6G). Of course, adding PR may require a smaller increase in the DAA dosage, rendering the DAA more tolerable.

Even where DAA-based treatment is likely to succeed, greater IFN-responsiveness would induce faster viral load decline and allow shorter treatment durations (Fig 6I and 6J). Using parameters representative of daclatasvir (Methods), we found that as $\phi_{1}^{p}$ decreased from ~0.1 to ~0.04, the treatment duration required for SVR dropped from ~12 weeks to ~8 weeks (Fig 6I and 6J). Although daclatasvir is not recommended for use as monotherapy, we use it here for illustration and because the NS5A region, the target of daclatasvir, is the one region where resistance testing may decide the choice of regimen [3]. Thus, individuals highly responsive to IFN present promising candidates for reducing DAA treatment durations. Indeed, we estimated that ~50% of the individuals treated with daclatasvir would achieve SVR in ~10 weeks and ~20% in ~8 weeks, durations expected to decrease further with DAA combinations, presenting a basis and a novel avenue for response-guided treatment.

## Discussion

DAAs, with >90% SVR rates in clinical trials, are bringing hope to the millions of chronically HCV infected individuals worldwide. In the present study, we elucidated a hypothesis underlying the unexpected positive correlation between the response elicited by DAAs and PR, which explains several confounding clinical observations and presents new potential avenues to improve DAA-based treatments. The hypothesis is that greater IFN-responsiveness restricts the replication space available for the virus, inhibiting the development of resistance to DAAs and improving treatment response. We developed a novel multiscale mathematical model to test this hypothesis. Analysis of a large body of clinical data using the model presented evidence in strong support of the hypothesis. The resulting causal relationship between responsiveness to PR and DAAs implied that increased responsiveness to PR could be exploited to prevent DAA failure and/or shorten the treatment duration, potentially positively impacting treatment response, tolerability, affordability and access.

Despite the high SVR rates they elicit, access to DAA-based treatments has seen limited so far; <1.3% of the ~150 million chronically HCV infected individuals are estimated to have received DAA-based treatment, with the proportion far smaller in resource-limited settings [52, 53]. To improve affordability and access, DAA-based treatments that would exert the most potent antiviral activity and/or patient subpopulations that would require the shortest durations are keenly being sought [54–59]. Our study informs these efforts by presenting new avenues to optimize DAA-based treatments. The standard strategy to avert DAA failure is to increase the genetic barrier to resistance by including more DAAs in the drug cocktail [12, 54]. In a recent set of studies, for instance, numerous DAA combinations were evaluated preclinically to identify the “best” candidates for clinical development and 3 DAA combinations were found to be more potent than 2 DAA combinations [54–56]. We suggest that an alternative strategy may often be feasible: improving IFN-responsiveness by adding IFN (or PR). Where additional DAAs remain inaccessible, especially in resource-limited settings, such a strategy may be useful. A previous modeling study also found that the efficacy of combining PR with a DAA significantly improved the treatment efficacy against DAA-resistant strains compared to the DAA alone [12]. Furthermore, our study quantified the gain in IFN-responsiveness due to standard PR dosage in patients with and without liver cirrhosis, representing a key potential step in personalizing the strategy. Response-guided treatment (RGT) is being considered now to define reduced treatment durations for select populations [27, 57, 58, 60, 61]. For instance, in a recent study, patients who achieved an ultra-rapid early viral load decline (plasma HCV RNA <500 copies/mL by day 2) were found to be cured with just 3 weeks of treatment [57]. Our prediction that individuals with high IFN-responsiveness are amenable to shorter treatment durations presents a much sought-after basis and a promising candidate population for reducing treatment durations, informing ongoing efforts to develop RGT protocols.

Personalizing treatment based on the avenues above requires estimation of the level of IFN-responsiveness of individuals. For a treatment-experienced individual, this may be achieved through analysis of viral load changes recorded during the previous PR treatment [17]. For a treatment-naive individual, short-term PR exposure and subsequent measurements of viral load may be necessary. Viral load changes as early as 24 h following the start of PR treatment have been argued to be good indicators of eventual response [62, 63]. Previous modeling studies have suggested a lead-in period of PR to assess the level of ongoing viral replication and the responsiveness to PR in order to decide optimal treatments [12, 64]. Developing such indicators to quantify the level of IFN-responsiveness, a promising future research direction based on our present study, would allow personalizing the course of DAA treatments also for treatment-naive individuals. Further, a correlation between IFN-responsiveness and the duration of DAA-based treatment required to achieve SVR would present a direct clinical test of our hypothesis.

Recent studies present further evidence in support of our hypothesis. In a study involving 240 chronic HCV patients treated with sofosbuvir and either daclatasvir or simeprevir for 12 weeks, slow responders, defined as those with detectable viremia at week 12, had a much higher representation of treatment (PR)-experienced patients than the overall population, *viz*., 82% versus 68%, indicating that IFN-free DAA treatments elicited slower viral load declines in individuals with poorer IFN-responsiveness [65]. Another study involving 216 patients treated with sofosbuvir and either daclatasvir or ledipasvir for 12 weeks found that baseline RAVs and treatment experience did not influence SVR in patients without cirrhosis but had a significant influence in patients with cirrhosis [66]. In yet another study [13], 6 of the 8 patients treated with daclatasvir and PR achieved SVR, of which 4 had RAVs detected pre-treatment, but had favourable IL28B genotypes (TT/GT) [37] and were treatment-naive or partial responders to prior IFN therapy. The 2 who failed treatment had unfavorable/partially favorable IL28B genotypes (GG/GT) and were null responders to prior IFN therapy. They experienced virological breakthrough due to the growth of RAVs although the RAVs were not detected pre-treatment. Earlier studies with the first generation DAAs provide further evidence. In a pooled study involving a large number of patients treated with boceprevir and PR, SVR rates were 78% and 76% in IFN-responders with and without baseline RAVs, respectively, whereas in poor IFN-responders, the corresponding SVR rates were 22% and 37% [67]. Similarly, with telaprevir, on-treatment virological failure rates were 1% in previous relapsers to PR, 19% in previous partial responders to PR, and 52% in previous null responders to PR [68]. Thus, in all the above cases, treatment failure was due to drug resistance, which did not depend on the pre-existence of RAVs but was facilitated by poor IFN-responsiveness. Conversely, strong IFN responses appeared to prevent the growth of RAVs and avert treatment failure. These findings are consistent with our model predictions.

Our study makes key advances in our understanding of HCV infection and treatment. To test the hypothesized causal relationship between responsiveness to IFN and DAAs, we had to construct a model that integrated phenomena across multiple length and time scales, starting from the cellular to the population level. Responsiveness to IFN manifests at the cellular level, defining the fraction of cells that can be rid of HCV by IFN. The consequence at the level of an infected individual is in restricting viral replication and evolution and hence improving responsiveness to DAAs. At the population level, this effect, given the distribution of IFN-responsiveness, determines SVR rates. Integration of phenomena across these scales into a single mathematical framework had not been accomplished thus far. Our model, by doing so, was able to capture the implications of variations at the cellular level, due to drugs, for instance, for the population-level treatment response. This allowed us to describe many clinical observations of which several had long remained unexplained, *viz*., 1) the percentage of infected individuals who spontaneously clear HCV, 2) the percentage of chronically infected individuals who exhibit a null response to PR, 3) the percentage of null responders to PR who respond to triple therapy with PR and a DAA such as telaprevir, and 4) the relationship between SVR rates in treatment-naive and treatment-experienced patients elicited by different treatments. A far more comprehensive view of HCV infection and treatment than earlier thus emerges.

The model we developed is complex. Yet, we only considered phenomena essential to establishing the causal relationship between IFN-responsiveness and DAA-based treatment outcomes. We thus ignored alternative mechanisms of DAA action [23, 69], specific intracellular viral replication events [11, 24], modes of synergy between IFN and DAAs [17, 51], and factors such as race, gender, viral genotype, and IL28B polymorphisms [1]. Furthermore, we did not estimate the IFN-responsiveness of an individual *a priori*. The key components of the IFN signaling network in cells have been identified [28], but variations in their levels and interactions across cells in an individual, which would determine the fraction of cells responsive to IFN, remain to be established. Finally, we assumed SVR to have been achieved when the viral load dropped below the “cure boundary” of 1 virion in the ~15 liters of fluid volume in an individual [5, 16]. With the new DAA combinations, some individuals with detectable viremia at the end of treatment have been found recently to achieve SVR [70]. The origins of this intriguing phenomenon, which may lead to the definition of a new cure boundary, remain poorly elucidated [71–73]. By employing the “stricter” cure boundary, our model yields conservative estimates of SVR rates.

## Methods

### Mathematical model

#### IFN-responsiveness and viral kinetics in an individual undergoing DAA-based treatment

We considered an individual chronically infected with HCV, subjected to a DAA-based treatment regimen. We constructed the following equations to describe the viral kinetics in the individual following the start of treatment when a single point mutation was adequate to confer resistance to the DAA:

$$\frac{dT_{i}}{dt}=\phi_{i}s+r_{T}T_{i}\left[1-\frac{\sum_{i=1}^{3}\left(T_{i}+\sum_{j=0}^{1}I_{i}^{j}\right)+N}{T_{\max}}\right]-T_{i}(1-\eta_{i})\left(\sum_{j=0}^{1}\beta_{j}V_{j}\right)-d_{T}T_{i};\;i\in \{1,2,3\}$$ (1)

$$\frac{dI_{i}^{j}}{dt}=r_{I}I_{i}^{j}\left[1-\frac{\sum_{i=1}^{3}\left(T_{i}+\sum_{k=0}^{1}I_{i}^{k}\right)+N}{T_{\max}}\right]+T_{i}(1-\eta_{i})\beta_{j}V_{j}-\delta I_{i}^{j};\;i\in \{1,2,3\},j\in \{0,1\}$$ (2)

$$\frac{dV_{0}}{dt}=(1-\mu )(1-\varepsilon_{DAA}^{0})p_{0}\sum_{i=1}^{3}(1-\varepsilon_{i})I_{i}^{0}-cV_{0}$$ (3)

$$\frac{dV_{1}}{dt}=\mu (1-\varepsilon_{DAA}^{0})p_{0}\sum_{i=1}^{3}(1-\varepsilon_{i})I_{i}^{0}+(1-\varepsilon_{DAA}^{1})p_{1}\sum_{i=1}^{3}(1-\varepsilon_{i})I_{i}^{1}-cV_{1}$$ (4)

Here, we classified uninfected target cells into 3 categories, denoted *T*_*i*_ with *i*ϵ{1,2,3}, based on the properties of the IFN signalling network in cells we identified earlier [17] (see Results). Broadly, IFN induces the expression of ISGs that suppress HCV, whereas HCV subverts the IFN response by blocking ISG translation. The resulting network, with the double negative feedback, exhibits bistability, *i*.*e*., two stable steady states, with HCV thriving in one and cleared by IFN in the other. The steady states define the 3 cell categories based on their IFN response phenotypes. Cells *T*_1_ admitted the first steady state alone and were refractory to IFN. IFN would prevent neither the infection of *T*_1_ nor viral production from them following their infection. Cells *T*_2_ admitted both the steady states and were bistable. Such cells are driven to the IFN-refractory state following their infection if the HCV RNA level crosses a threshold before exposure to IFN [17, 30]. Else, they are driven to the IFN-responsive state. Thus, as an approximation, we assumed that IFN would prevent infection of *T*_2_ if they were exposed to IFN before the virus, but fail to prevent viral production from such cells once infected. Cells *T*_3_ admitted the second steady state alone and were sensitive to IFN. IFN would thus prevent infection of *T*_3_ and if infected block viral production from such cells.

*s* was the production rate of target cells, and *ϕ*_*i*_ was the fraction of the cells produced that was of type *T*_*i*_. In the absence of infection and when cell proliferation is small, *ϕ*_*i*_ equals the fraction of the target cells that is of type *T*_*i*_. *ϕ*_1_ was thus the fraction of cells refractory to IFN and quantified the level of IFN-responsiveness of an individual. Lower values of *ϕ*_1_ represented greater IFN-responsiveness. We set $\phi_{1}=\phi_{1}^{p}$ pre-treatment and during IFN-free treatment and $\phi_{1}=\phi_{1}^{t}$ during IFN-containing treatment. The cells died with the rate constant *d*_*T*_. The cells were also lost due to infection by free virions.

*V*_0_ and *V*_1_ were the wild-type and RAV viral populations, respectively. When a cell *T*_*i*_ was infected by a virion *V*_*j*_, it gave rise to an infected cell of the type $I_{i}^{j}$. The infectivity of the virions *V*_*j*_ was denoted *β*_*j*_. Further, successful infection of the cell *T*_*i*_ was blocked by IFN with efficacy *η*_*i*_. From the description above, it followed that *η*_1_ = 0 and *η*_2_ = *η*_3_ = 1. Target cells and infected cells proliferated with rate constants *r*_*T*_ and *r*_*I*_, respectively, constrained by a logistic term with carrying capacity *T*_max_. *N* represented the population of target cells not susceptible to infection, due, for instance, to the lack of adequate entry receptors [74]. Infected cells were lost with the rate constant *δ*.

*p*_*j*_ represented the per cell production rate of virions from cells $I_{i}^{j}$. IFN constrained this production with effectiveness *ε*_*i*_, dependent on the cell phenotype. Again, from the description above, *ε*_1_ = *ε*_2_ = 0 and *ε*_3_ = 1.

DAAs limited viral production from all cells with effectiveness $\varepsilon_{DAA}^{j}$, dependent on the viral variant. Mutations arose randomly and compromised DAA activity. The mutations came with a fitness cost to the virus, determined here by lower values of *β*_*j*_ and/or *p*_*j*_. Specifically, mutation at the rate *μ* allowed the production of *V*_1_ from cells $I_{i}^{0}$, infected with *V*_0_. We ignored back mutation of *V*_1_ to *V*_0_ (e.g., see [12, 22, 75]). Free virions were cleared with the rate constant *c*.

A generalization of the formalism to multiple resistance loci is presented in S2 Text.

#### Distribution of IFN-responsiveness across treatment-naive and -experienced individuals

We assumed that $\phi_{1}^{p}$ was log-normally distributed with parameters *ν* and *σ* across individuals in a treatment-naive HCV-infected population. The density function for $\phi_{1}^{p}$ was thus

$$f\left(\phi_{1}^{p}\right)=\frac{A}{\sqrt{2\pi }\sigma \phi_{1}^{p}}\exp\left(-\frac{{\left(\ln\phi_{1}^{p}-\nu \right)}^{2}}{2\sigma^{2}}\right);\;0\leq \phi_{1}^{p}\leq 1,$$ (5)

where $A=1/\int_{0}^{1}\frac{1}{\sqrt{2\pi }\sigma \phi }\exp\left(-\frac{{\left(\ln\phi -\nu \right)}^{2}}{2\sigma^{2}}\right)d\phi$ ensured that $\int_{0}^{1}f\left(\phi \right)d\phi =1$.

When $\phi_{1}^{p}<\phi_{1}^{c}\approx d_{T}c\delta /\beta sp_{0}$ (Eq. (S1.10)), the model above (Eqs (1)–(4)) resulted in vanishing set-point viral load pre-treatment, potentially representing individuals who spontaneously clear HCV [17, 45]. The distribution of $\phi_{1}^{p}$ in chronically infected, treatment-naive individuals thus became the truncated log-normal,

$$f_{naive}(\phi_{1}^{p})=\frac{f(\phi_{1}^{p})}{\int_{\phi_{1}^{c}}^{1}f\left(\phi \right)d\phi };\;\phi_{1}^{c}<\phi_{1}^{p}\leq 1.$$ (6)

We denoted by *ϕ*_*null*_ the value of $\phi_{1}^{p}$ above which PR treatment elicited a null response. Null response, or non-response, was defined as having occurred when <2 log_10_ decline in viral load resulted from 12 weeks of treatment. Given *ϕ*_*null*_, the density function of $\phi_{1}^{p}$ in null responders,

$$f_{null}\left(\phi_{1}^{p}\right)=\frac{f_{naive}\left(\phi_{1}^{p}\right)}{\int_{\phi_{null}}^{1}f_{naive}\left(\phi \right)d\phi };\;\phi_{null}\leq \phi_{1}^{p}\leq 1.$$ (7)

#### Rates of spontaneous clearance, null response, and SVR in populations

The fraction, *CL*, of HCV infected individuals who spontaneously clear the infection would include all those with $\phi_{1}^{p}<\phi_{1}^{c}$; i.e.,

$$CL=\int_{0}^{\phi_{1}^{c}}f\left(\phi_{1}^{p}\right)d\phi_{1}^{p}.$$ (8)

We defined $\phi_{SVR}^{DAA}$ as the highest value of $\phi_{1}^{p}$ for which a treatment-naive individual would achieve SVR when subjected to a particular IFN-free DAA regimen. SVR was defined as achieved when the viral load reached a value <1 virion in the 15 liters of fluid volume in an individual by the end of treatment [5, 16]. The fraction of the population treated with the regimen that would achieve SVR, which we called $SVR_{naive}^{DAA}$, would therefore be

$$SVR_{naive}^{DAA}=\int_{\phi_{1}^{c}}^{\phi_{SVR}^{DAA}}f_{naive}\left(\phi_{1}^{p}\right)d\phi_{1}^{p}.$$ (9)

Similarly, if $\phi_{SVR}^{PR+DAA}$ was the highest value of $\phi_{1}^{p}$ for which SVR was achieved with the above DAA regimen combined with PR, the fraction of a treatment-naive population that would achieve SVR with this combination would be

$$SVR_{naive}^{PR+DAA}=\int_{\phi_{1}^{c}}^{\phi_{SVR}^{PR+DAA}}f_{naive}\left(\phi_{1}^{p}\right)d\phi_{1}^{p}.$$ (10)

The fraction of a treatment-naive population that would exhibit a null response to PR, denoted $NULL_{naive}^{PR}$, would be

$$NULL_{naive}^{PR}=\int_{\phi_{null}}^{1}f_{naive}\left(\phi_{1}^{p}\right)d\phi_{1}^{p}.$$ (11)

Of the prior null responders to PR, the fraction that would respond to the above DAA would be

$$SVR_{null}^{DAA}=\int_{\phi_{null}}^{\phi_{SVR}^{DAA}}f_{null}\left(\phi_{1}^{p}\right)d\phi_{1}^{p}$$ (12)

and, finally, to the PR+DAA combination would be

$$SVR_{null}^{PR+DAA}=\int_{\phi_{null}}^{\phi_{SVR}^{PR+DAA}}f_{null}\left(\phi_{1}^{p}\right)d\phi_{1}^{p}.$$ (13)

#### The distributions and threshold values of $\phi_{1}^{p}$ and the linkage between individual- and population-level models

We identified the various threshold values of $\phi_{1}^{p}$ mentioned above as follows. We first solved our viral dynamics model (Eqs (1)–(4)) using parameter values listed in S5 Table and identified $\phi_{1}^{c}$. Next, we recognized that the baseline viral load when $\phi_{1}^{p}>\phi_{1}^{c}$ is $V\approx \frac{\phi_{1}^{p}sp_{0}}{c\delta }-\frac{d_{T}}{\beta_{0}}\approx \frac{\phi_{1}^{p}sp_{0}}{c\delta }$ (see Eq. (S1.10) in S1 Text and the parameter estimates in S5 Table), which has the maximum, $V_{\max}\approx \frac{sp_{0}}{c\delta }$ when $\phi_{1}^{p}=1$. It followed that $\phi_{1}^{p}\approx V/V_{\max}$. We therefore fit Eq (6) to the reported distribution of *V*/*V*_max_ values [44] and obtained *ν* and *σ*.

For any DAA, $\phi_{SVR}^{DAA}$ can be estimated by solving Eqs (1)–(4) for different values of $\phi_{1}^{p}=\phi_{1}^{t}$ and with $\varepsilon_{DAA}^{j}$ corresponding to the effectiveness of the DAA against wild-type and RAV strains, and identifying the maximum value of $\phi_{1}^{p}$ for which SVR is achieved. For IFN-containing regimens, Eqs (1)–(4) cannot be solved to obtain $\phi_{SVR}^{PR+DAA}$ because the relationship between $\phi_{1}^{t}$ when $\phi_{1}^{p}$ is not known *a priori*. We therefore employed population-level observations of $SVR_{naive}^{PR+DAA}$ for telaprevir [48] and solved Eq (8) for $\phi_{SVR}^{PR+DAA}$. This yielded an estimate of $\Delta \phi =\phi_{SVR}^{PR+DAA}-\phi_{SVR}^{DAA}$ for telaprevir. Because the dynamics during treatment is dictated essentially by $\phi_{1}^{t}$ (see Results), it followed that $\phi_{1}^{t}\approx \phi_{SVR}^{DAA}$ when $\phi_{1}^{p}=\phi_{SVR}^{PR+DAA}$, defining the threshold for SVR. Δ*ϕ* is therefore a measure of the increase in IFN-responsiveness due to the addition of IFN as part of PR treatment. Further, because synergy between telaprevir and PR appears small (see Results), Δ*ϕ* represents the increase in IFN-responsiveness brought about independently by PR.

We validated the resulting estimate of Δ*ϕ* as follows. Δ*ϕ* would depend on the extent of increase in the IFN concentration above the endogenous level because of treatment. For standard PR dosage, Δ*ϕ* may thus be assumed to apply across individuals. (We recognized that this assumption would fail when $\phi_{1}^{p}$ is small and where the influence of IFN is expected to saturate. A small $\phi_{1}^{p}$, however, would amount to an already IFN-sensitive individual and to whom addition of PR is likely to be unnecessary.) Δ*ϕ* may differ between individuals with cirrhotic and non-cirrhotic livers, which we address below. Using the above estimate of Δ*ϕ*, we solved Eqs (1)–(4) for different values of $\phi_{1}^{p}$ and $\phi_{1}^{t}=\phi_{1}^{p}-\Delta \phi$ and identified *ϕ*_*null*_ as the lowest $\phi_{1}^{p}$ that yielded a null response. With the resulting value of *ϕ*_*null*_, we estimated $NULL_{naive}^{PR}$ using Eq (11) and $SVR_{null}^{PR+DAA}$ using Eq (13) and compared the estimates with observations [48, 49].

With the distributions and all the threshold values of $\phi_{1}^{p}$ thus identified, Eqs (1)–(13) presented a model that could predict the outcomes of DAA-based treatments at the individual and the population level.

### Solution of model equations

#### Viral kinetics

In our model, DAAs are distinguished by their efficacies, $\varepsilon_{DAA}^{j}$, against sensitive and resistant strains as well as the relative fitness of the respective RAVs, defined by *γ*. To describe the scenario pre-treatment in different individuals, we set $\varepsilon_{DAA}^{j}=0$ and solved Eqs (1)–(4) for steady state to obtain the set point viral load and the frequencies and populations of RAVs as functions of $\phi_{1}^{p}$. We also derived analytical approximations of these steady state quantities (S1 Text). Using the above steady state as the initial condition, we solved Eqs (1)–(4) with $\varepsilon_{DAA}^{j}>0$ and $\phi_{1}^{t}=\phi_{1}^{p}$ to describe viral load changes following the onset of IFN-free DAA-based treatment. For IFN-containing regimens, we followed the same procedure but with $\phi_{1}^{t}<\phi_{1}^{p}$.

#### Parameter estimates and sensitivity

Model equations were solved using parameter values representative of HCV infection *in vivo*, listed in S5 Table. Sensitivity of model predictions to parameter values was tested by computing the partial rank correlation coefficients (PRCC) [76].

#### SVR rates

For given values of $\varepsilon_{DAA}^{j}$ and *γ*, representing a particular DAA or DAA combination, Eqs (1)–(13) were solved to obtain estimates of SVR rates in treatment-naïve and treatment-experienced patients. The values of $\varepsilon_{DAA}^{j}$ and *γ* have not been characterized for most DAAs. *γ* and the ratio $\varepsilon_{DAA}^{1}/\varepsilon_{DAA}^{0}$, however, must lie between 0 and 1 for all DAAs. We therefore varied each of these quantities over the entire range of values from 0 to 1 and computed SVR rates, where each combination of these parameter values would represent a different DAA-based treatment. For each combination, we first estimated $\phi_{SVR}^{DAA}$ by solving Eqs (1)–(4). Next, using Δ*ϕ* obtained above, we estimated $\phi_{SVR}^{PR+DAA}=\Delta \phi +\phi_{SVR}^{DAA}$. All the other model parameters and quantities remained independent of the DAAs. Using Eqs (5)–(13), we estimated SVRs in treatment-naïve and treatment-experienced individuals. Repeating this with other parameter combinations yielded a relationship between SVR rates in the two patient populations, which we compared with clinical data. We also derived an analytical expression for the latter relationship below.

The numerical solutions were implemented in MATLAB.

### Relationship between SVR rates in treatment-naïve and treatment-experienced patients

We let *SVR*_*naive*_ and *SVR*_*null*_ be the response to any given DAA-based treatment in a treatment-naïve and a previous null responder population, respectively. We derived an analytical expression linking *SVR*_*naive*_ and *SVR*_*null*_ as follows. From our model above, it followed that

$$SVR_{naive}^{}=\int_{\phi_{1}^{c}}^{\phi_{SVR}^{}}f_{naive}\left(\phi_{1}^{p}\right)d\phi_{1}^{p}$$ (14)

and

$$SVR_{null}^{}=\int_{\phi_{null}}^{\phi_{SVR}^{}}f_{null}\left(\phi_{1}^{p}\right)d\phi_{1}^{p}.$$ (15)

Using the relationship between $f_{naive}\left(\phi_{1}^{p}\right)$ and $f_{null}\left(\phi_{1}^{p}\right)$ (Eq (7)) in Eq (15) yielded

$$SVR_{null}^{}=\frac{\int_{\phi_{null}}^{\phi_{SVR}^{}}f_{naive}\left(\phi_{1}^{p}\right)d\phi_{1}^{p}}{\int_{\phi_{null}}^{1}f_{naive}\left(\phi_{1}^{p}\right)d\phi_{1}^{p}}=\frac{1}{NULL_{naive}^{PR}}\int_{\phi_{null}}^{\phi_{SVR}^{}}f_{naive}\left(\phi_{1}^{p}\right)d\phi_{1}^{p}$$ (16)

where $NULL_{naive}^{PR}$ is defined in Eq (11). We next rearranged the integral in Eq (16) as

$$\int_{\phi_{null}}^{\phi_{SVR}^{}}f_{naive}\left(\phi_{1}^{p}\right)d\phi_{1}^{p}=\int_{\phi_{null}}^{\phi_{1}^{c}}f_{naive}\left(\phi_{1}^{p}\right)d\phi_{1}^{p}+\int_{\phi_{1}^{c}}^{\phi_{SVR}^{}}f_{naive}\left(\phi_{1}^{p}\right)d\phi_{1}^{p}=-(1-NULL_{naive}^{PR})+SVR_{naive}$$ (17)

and obtained upon combining with Eq (16) and rearranging,

$$SVR_{naive}=1-NULL_{naive}^{PR}+NULL_{naive}^{PR}SVR_{null}^{}.$$ (18)

We fit the expression above to clinical data using the NLINFIT algorithm in MATLAB.

### Data from clinical trials

We examined reports of all clinical trials with DAA-based treatments and considered those treatments for which SVR data on both treatment-naïve and treatment-experienced individuals was available. The resulting data is summarized in Table 1 and S1 Table. We also classified the patient populations into categories with and without liver cirrhosis. We performed statistical tests to ascertain the difference in the SVR rates between treatment-naïve and treatment-experienced individuals for specific treatments as well as when data for all the treatments considered were combined. We compared the predictions above of SVR rates with the data from clinical trials.

### IFN-responsiveness of cirrhotic and non-cirrhotic patients

Next, we considered SVR data on patients with and without liver cirrhosis separately. Predicting this data using our model was not possible because Δ*ϕ* and *ϕ*_*null*_ in these populations were not known. We therefore fit Eq (18) to the two data sets separately using $NULL_{naive}^{PR}$ as an adjustable parameter. The distribution of baseline viral loads is not hugely different between the two populations, although mean viral loads were somewhat smaller in patients with liver cirrhosis [44]. Using Eq (11) and the best-fit estimates of $NULL_{naive}^{PR}$, we obtained estimates of *ϕ*_*null*_ in the two populations. Finally, we solved our model of viral dynamics (Eqs (1)–(4)) with $\phi_{1}^{p}=\phi_{null}$ for different values of $\phi_{1}^{t}=\phi_{1}^{p}-\Delta \phi$ and identified the highest value of $\phi_{1}^{t}$ that yielded a null response. The resulting values of $\Delta \phi =\phi_{1}^{p}-\phi_{1}^{t}$ provided estimates of the extent of increase in IFN-responsiveness due to standard PR treatment in cirrhotic and non-cirrhotic patients, respectively, which allowed recommendation of strategies to improve treatments in these subpopulations.

### Estimation of required treatment duration

Finally, we calculated the required duration of treatment to achieve SVR for different $\phi_{1}^{p}$ and *γ*_*t*_. We chose parameters representative of daclatasvir as follows. The EC_50_ of daclatasvir against the wild-type [77] and the RAV [78] were 17.28 pM and 32346.26 pM, respectively. (The molecular weight of daclatasvir is 738.89 g/mol.) Using the pharmacokinetic parameters of daclatasvir [79], the peak and trough plasma concentrations, *C*_max_ = 1726.4 ng/ml and *C*_min_ = 254.6 ng/ml, the dosing interval of 1 d, and the time to reach peak plasma concentration of 1 h, we calculated the average efficacy of daclatasvir against the wild-type and the RAV, following the procedure outlined earlier [77], to be 0.99998 and 0.709, respectively. With these values, we solved our model of viral dynamics (Eqs (1)–(4)) for different values of $\phi_{1}^{p}$ and the intrinsic fitness of the RAV, *γ*, and estimated the duration of treatment required to achieve SVR. For the common RAV Y93H, *γ* = 0.5751 [77]. Using this value of *γ* and the distribution of $\phi_{1}^{p}$ in treatment naïve individuals (Eq (6)), we estimated the fraction of individuals treated who would achieve SVR within a defined treatment duration.

## Supporting information

S1 FigSensitivity analysis of the viral kinetic model.Partial rank correlation coefficients (PRCCs) indicating the sensitivity of our model predictions (Eqs (1)–(4) and (S2.1)-(S2.4)) of **(A)** single mutant population pre-treatment, **(B)** single mutant population during DAA treatment, **(C)** double mutant population pre-treatment, and **(D)** double mutant population during DAA treatment to variations in model parameter values. The model is considered sensitive to parameters with PRCCs significantly different from the dummy. Thus, the model is sensitive to $\mu ,\gamma ,\phi_{1}^{p},s,p_{0},c,$ and *δ* pre-treatment, in agreement with the parameters defining the mutant population in the analytical approximation in Eq. (S1.11), and additionally to the drug efficacies, $\varepsilon_{DAA}^{0}$ and $\varepsilon_{DAA}^{1}$, during treatment. For these calculations, we adapted the MATLAB codes available on Dr. Denise Kirschner’s website (http://malthus.micro.med.umich.edu/lab/usadata).(TIF)

S2 FigPhase diagrams indicating response to IFN-free DAA treatments.The level of IFN-refractoriness, $\phi_{1}^{p}$, and the relative fitness of the RAV during treatment, *γ*_*t*_, that lead to SVR (dark blue) or treatment failure due to virological breakthrough by the RAV (light blue), wild-type (green), or both (brown) when **(A)** $\varepsilon_{0}^{DAA}=0.70$, **(B)** $\varepsilon_{0}^{DAA}=0.80$, **(C)** $\varepsilon_{0}^{DAA}=0.90$, **(D)** $\varepsilon_{0}^{DAA}=0.99$. Here, *γ* = 0.5. The other parameters are the same as in Fig 3.(TIF)

S3 FigPhase diagrams indicating response to PR+DAA treatments.IFN-refractoriness pre- and during treatment, $\phi_{1}^{p}$ and $\phi_{1}^{t}$, leading to SVR (dark blue), or virological breakthrough by RAV (light blue), wild-type (green), or both (brown) when **(A)** $\varepsilon_{0}^{DAA}=0.99,\varepsilon_{1}^{DAA}=0.1$; **(B)** $\varepsilon_{0}^{DAA}=0.99,\varepsilon_{1}^{DAA}=0.2$; **(C)** $\varepsilon_{0}^{DAA}=0.99,\varepsilon_{1}^{DAA}=0.3$; **(D)** $\varepsilon_{0}^{DAA}=0.95,\varepsilon_{1}^{DAA}=0.1$; **(E)** $\varepsilon_{0}^{DAA}=0.95,\varepsilon_{1}^{DAA}=0.2$ and **(F)** $\varepsilon_{0}^{DAA}=0.95,\varepsilon_{1}^{DAA}=0.3$. Here, *γ* = 0.1. The other parameters are the same as in Fig 4.(TIF)

S1 Table Response to DAA-based treatments. SVR rates elicited by various IFN-free and IFN-containing DAA combinations in treatment-naïve and prior null responders to PR from recent clinical trials. The treated population size is indicated in brackets. The significance of the difference in the SVR rates in the two populations is computed using the χ^2^ and the Fisher’s exact tests. The HCV genotype and whether the patients had liver cirrhosis is indicated. Data from all trials involving a particular treatment regimen are combined for the statistical analysis.
(DOCX)

S2 Table Response to DAA-based treatments in patients with liver cirrhosis. The datasets in S1 Table that consider patients with liver cirrhosis alone are summarized.
(DOCX)

S3 Table Response to DAA-based treatments in patients without liver cirrhosis. The datasets in S1 Table that consider patients without liver cirrhosis alone are summarized.
(DOCX)

S4 Table Response to DAA-based treatments from studies that do not distinguish between patients with and without cirrhosis. The datasets in S1 Table that consider patients without distinction in cirrhosis are summarized.
(DOCX)

S5 Table Model parameters. Definitions of model parameters and their typical values employed. Variations are mentioned in the text.
(DOCX)

S1 Text Analytical approximation of the pre-treatment steady state with a single resistance locus. (DOCX)

S2 Text Model formulation with multiple resistance loci. (DOCX)

S3 Text Analytical approximation of the pre-treatment steady state with two or more resistance loci. (DOCX)
