# Supplementary figures and images for: Modelling how responsiveness to interferon improves interferon-free treatment of hepatitis C virus infection
Source: PLoS Comput Biol. 2018 Jul 12;14(7):e1006335. doi: 10.1371/journal.pcbi.1006335 (PMC6057683; doi:10.1371/journal.pcbi.1006335)

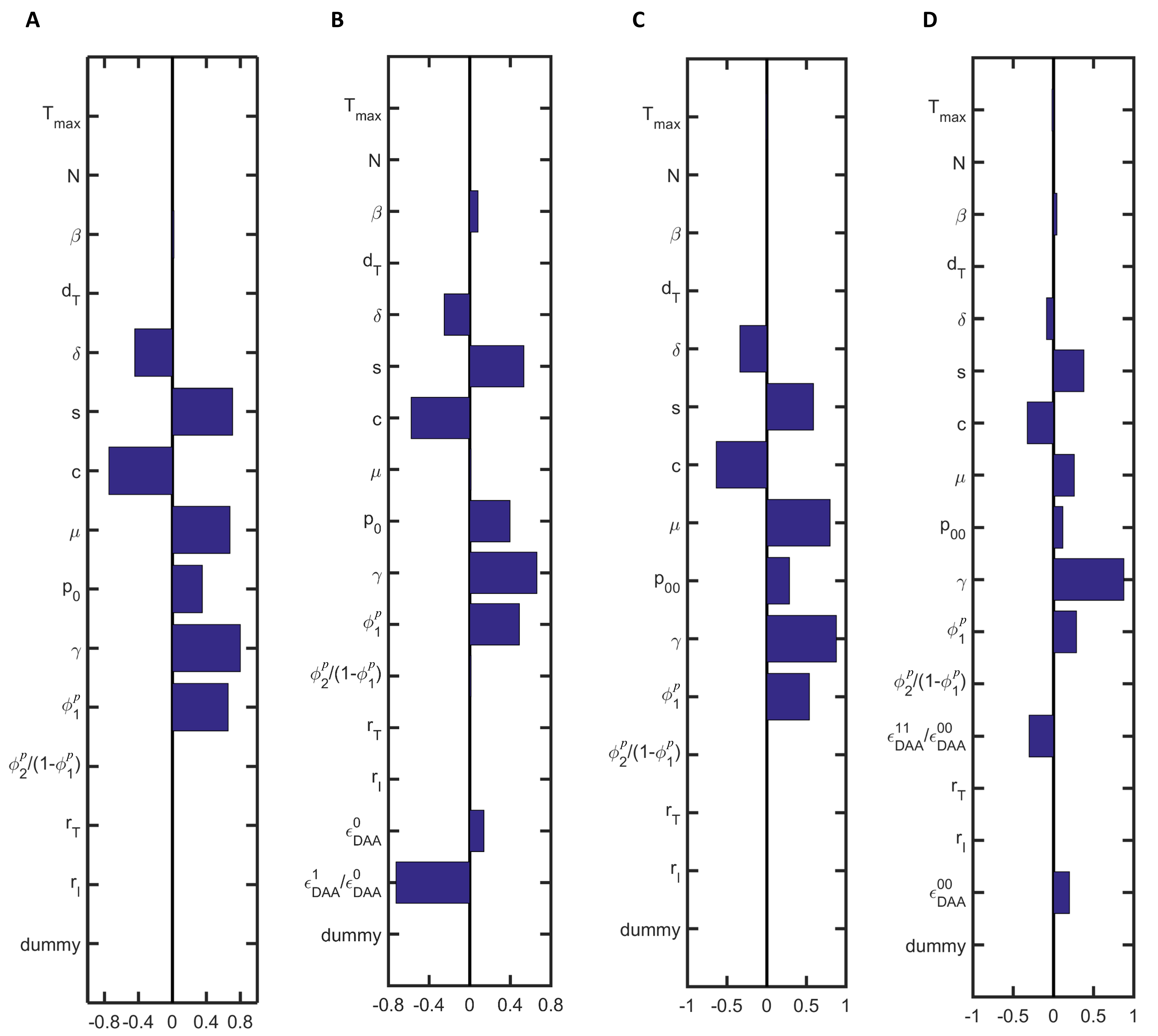

Supplement: S1 Fig — Partial rank correlation coefficients (PRCCs) indicating the sensitivity of our model predictions (Eqs (1)–(4) and (S2.1)-(S2.4)) of (A) single mutant population pre-treatment, (B) single mutant population during DAA treatment, (C) double mutant population pre-treatment, and (D) double mutant population during DAA treatment to variations in model parameter values. The model is considered sensitive to parameters with PRCCs significantly different from the dummy. Thus, the model is sensitive to μ,γ,ϕ1p,s,p0,c, and δ pre-treatment, in agreement with the parameters defining the mutant population in the analytical approximation in Eq. (S1.11), and additionally to the drug efficacies, εDAA0 and εDAA1, during treatment. For these calculations, we adapted the MATLAB codes available on Dr. Denise Kirschner’s website (http://malthus.micro.med.umich.edu/lab/usadata). (TIF) [file pcbi.1006335.s001.tif]

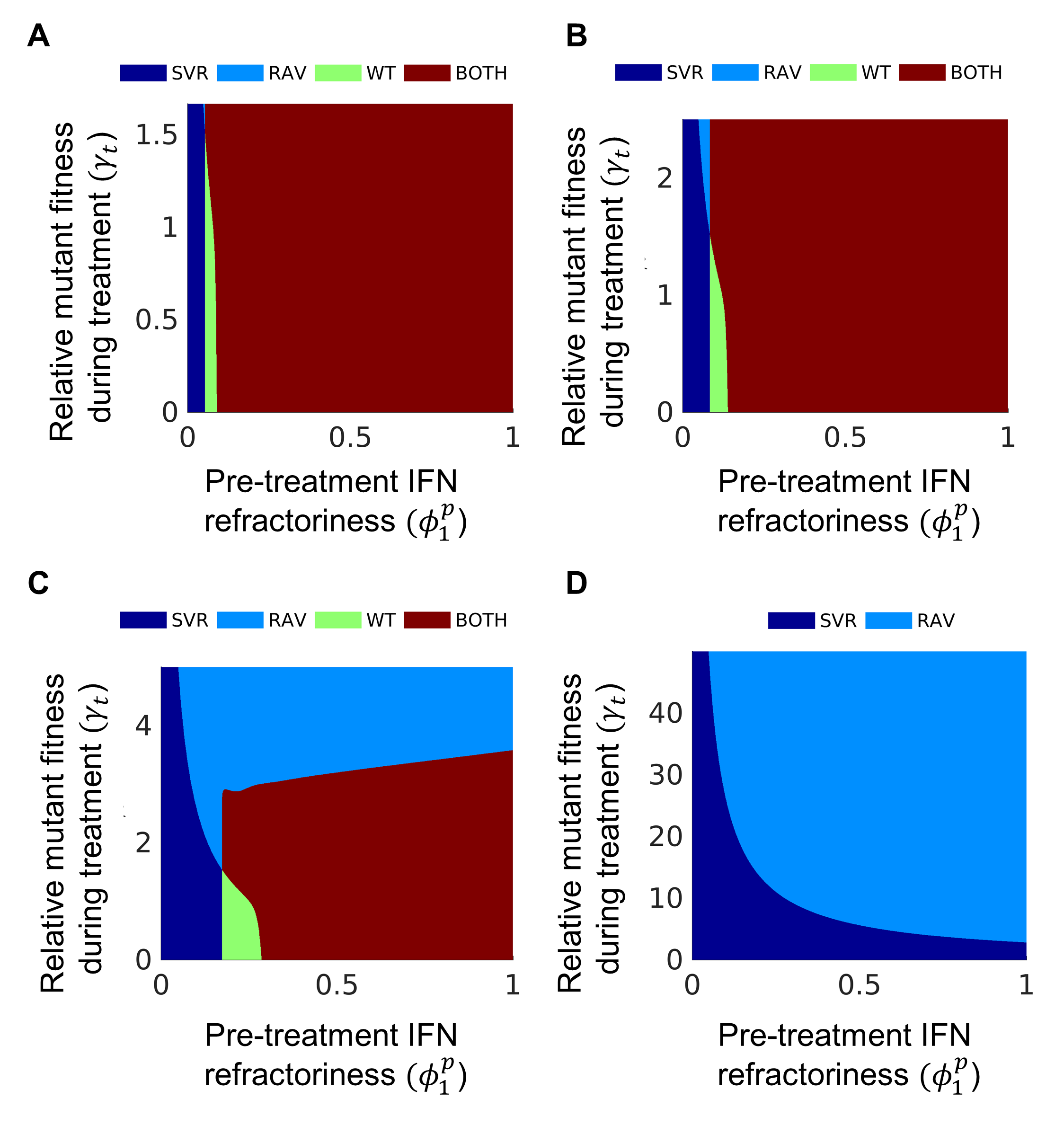

Supplement: S2 Fig — The level of IFN-refractoriness, ϕ1p, and the relative fitness of the RAV during treatment, γt, that lead to SVR (dark blue) or treatment failure due to virological breakthrough by the RAV (light blue), wild-type (green), or both (brown) when (A) ε0DAA=0.70, (B) ε0DAA=0.80, (C) ε0DAA=0.90, (D) ε0DAA=0.99. Here, γ = 0.5. The other parameters are the same as in Fig 3. (TIF) [file pcbi.1006335.s002.tif]

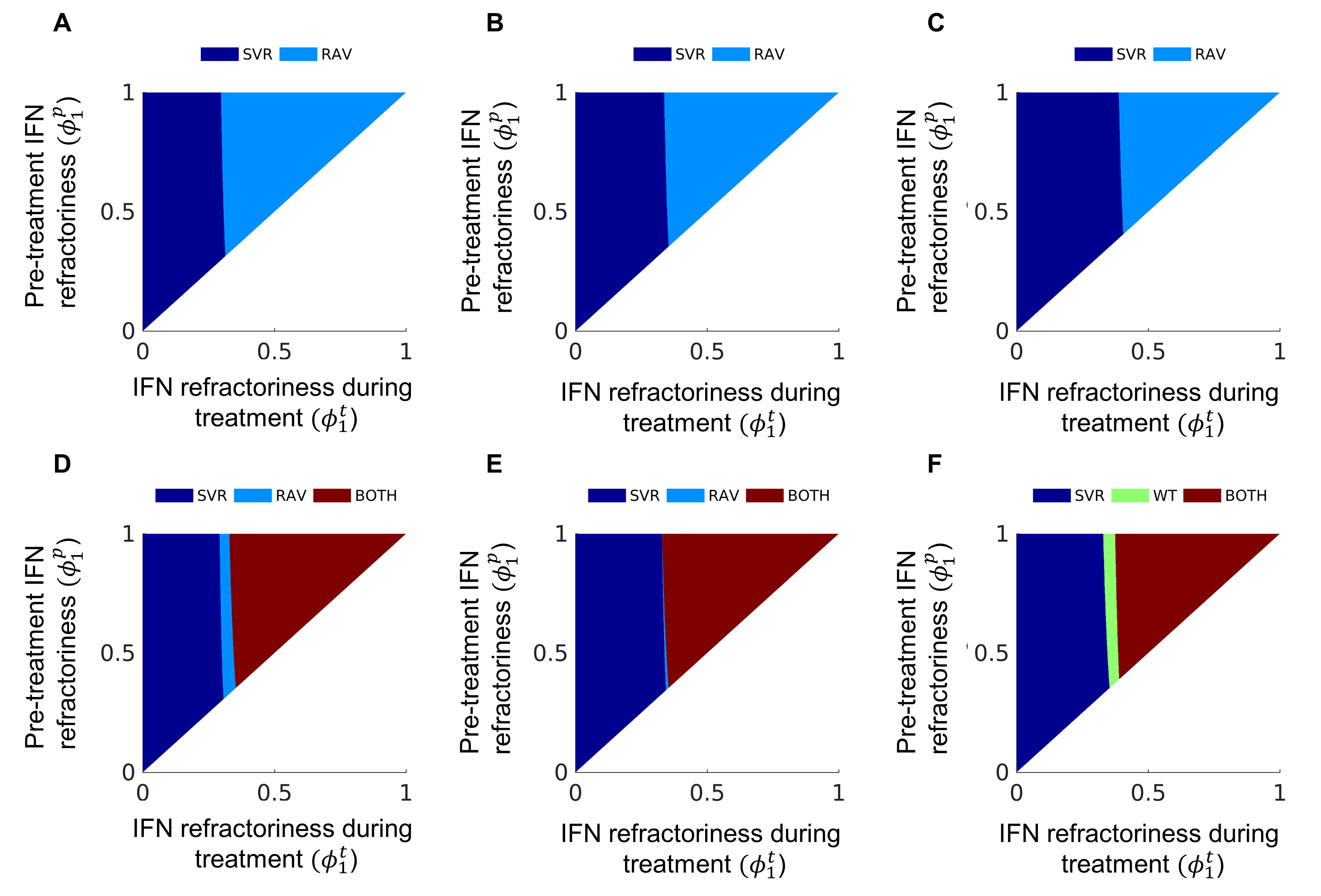

Supplement: S3 Fig — IFN-refractoriness pre- and during treatment, ϕ1p and ϕ1t, leading to SVR (dark blue), or virological breakthrough by RAV (light blue), wild-type (green), or both (brown) when (A) ε0DAA=0.99,ε1DAA=0.1; (B) ε0DAA=0.99,ε1DAA=0.2; (C) ε0DAA=0.99,ε1DAA=0.3; (D) ε0DAA=0.95,ε1DAA=0.1; (E) ε0DAA=0.95,ε1DAA=0.2 and (F) ε0DAA=0.95,ε1DAA=0.3. Here, γ = 0.1. The other parameters are the same as in Fig 4. (TIF) [file pcbi.1006335.s003.tif]
