# Supplementary material for: Modelling how responsiveness to interferon improves interferon-free treatment of hepatitis C virus infection
Source: PLoS Comput Biol. 2018 Jul 12;14(7):e1006335. doi: 10.1371/journal.pcbi.1006335 (PMC6057683; doi:10.1371/journal.pcbi.1006335)
Supplement: S1 Table — SVR rates elicited by various IFN-free and IFN-containing DAA combinations in treatment-naïve and prior null responders to PR from recent clinical trials. The treated population size is indicated in brackets. The significance of the difference in the SVR rates in the two populations is computed using the χ2 and the Fisher’s exact tests. The HCV genotype and whether the patients had liver cirrhosis is indicated. Data from all trials involving a particular treatment regimen are combined for the statistical analysis. (DOCX) [file pcbi.1006335.s004.docx]

**S1 Table. Response to DAA-based treatments.** SVR rates elicited by various IFN-free and IFN-containing DAA combinations in treatment-naïve and prior null responders to PR from recent clinical trials. The treated population size is indicated in brackets. The significance of the difference in the SVR rates in the two populations is computed using the χ^2^ and the Fisher’s exact tests. The HCV genotype and whether the patients had liver cirrhosis is indicated. Data from all trials involving a particular treatment regimen are combined for the statistical analysis.

| **Regimen** | **Genotype** | **Cirrhosis** | **% SVR (N)** | | **P-value** | | **Trial name/stage** | **Ref.** |
| --- | --- | --- | --- | --- | --- | --- | --- | --- |
|  |  |  | **Naïve** | **Null** | **χ^2^** | **Fisher** |  |  |
| Telaprevir + PegIFNα/RBV for 12w and PegIFNα/RBV for 12w to 48w | 1 | nd | 78.5 (363) |  |  |  | ADVANCE, phase 3 | [1] |
|  | 1 | nd | 74.1 (540) |  |  |  | ILLUMINATE, phase 3 | [1] |
|  | 1 | nd | 74.3 (369) |  |  |  | OPTIMIZE, phase3 | [1] |
|  | 1 | nd |  | 32 (147) |  |  | REALIZE, phase 3 | [1] |
|  | 1 | nd | 75.4 (1272) | 32 (147) | 5.17×10^-28^ | 3.54×10^-25^ | Combined |  |
|  | 1a | no | 82.4 (34) |  |  |  | MALACHITE-I, phase 3 | [2] |
|  | 1b | no | 78 (41) |  |  |  | MALACHITE-I, phase 3 | [2] |
|  | 1 | no |  | 56.5 (23) |  |  | MALACHITE-II, phase 3 | [2] |
|  | 1 | no |  | 50 (190) |  |  | ATTAIN, phase 3 | [3] |
|  | 1 | no | 62.2 (111) |  |  |  | Phase 3 | [4] |
|  | 1 | no | 77.8 (315) |  |  |  | OPTIMIZE, phase 3 | [5] |
|  | 1 | no | 61.8 (440) |  |  |  | HCV-TARGET, phase 3 | [6] |
|  | 1 | no | 68.7 (941) | 50.7 (213) | 6.70×10^-7^ | 1.46×10^-6^ | Combined |  |
|  | 1 | yes | 53.7 (54) |  |  |  | OPTIMIZE, phase 3 | [5] |
|  | 1 | yes | 43.5 (237) |  |  |  | HCV-TARGET, phase 3 | [6] |
|  | 1 | yes |  | 31.3 (48) |  |  | ATTAIN, phase 3 | [7] |
|  | 1 | yes |  | 19.4 (31) |  |  | ANRS CO20, phase 3 | [8] |
|  | 1 | yes | 45.4 (291) | 26.6 (79) | 2.65×10^-3^ | 2.94×10^-3^ | Combined |  |
| PegIFNα/RBV for 4w and boceprevir + PegIFNα/RBV for 44w | 1 | no | 76.3 (97) |  |  |  | SPRINT-1, phase 2 | [7] |
|  | 1 | no | 67.4 (331) |  |  |  | SPRINT-2, phase 2 | [9] |
|  | 1 | no | 59.2 (147) |  |  |  | HCV-TARGET, phase 3 | [6] |
|  | 1 | no |  | 40.8 (49) |  |  | PROVIDE, phase 3 | [10] |
|  | 1 | no | 64.1 (604) |  |  |  | Protocol 6086, phase 3 | [11] |
|  | 1 | no |  | 47.2 (36) |  |  | P05685AM2, phase 3 | [12] |
|  | 1 | no | 65.4 (1179) | 43.5 (85) | 5.03×10^-5^ | 9.24×10^-5^ | Combined |  |
|  | 1 | yes | 41.7 (24) |  |  |  | SPRINT-2, phase 2 | [9] |
|  | 1 | yes | 50 (6) |  |  |  | SPRINT-1, phase 2 | [7] |
|  | 1 | yes | 55 (60) |  |  |  | Protocol 6086, phase 3 | [11] |
|  | 1 | yes | 32 (50) |  |  |  | HCV-TARGET, phase 3 | [6] |
|  | 1 | yes |  | 0 (10) |  |  | ANRS CO20, phase 3 | [8] |
|  | 1 | yes | 44.3 (140) | 1 (10) | 6.01×10^-3^ | 5.48×10^-3^ | Combined |  |
| Simeprevir + PegIFNα/RBV for 12w and PegIFNα/RBV for 12w | 1 | no | 80.5 (77) |  |  |  | PILLAR, phase 2b | [13] |
|  | 1 | no | 88.6 (123) |  |  |  | CONCERTO-1, phase 3 | [14] |
|  | 1 | no |  | 52.8 (53) |  |  | CONCERTO-2, phase 3 | [15] |
|  | 1 | no | 91.7 (24) | 38.5 (26) |  |  | CONCERTO-4, phase 3 | [16] |
|  | 1 | no | 82.1 (229) |  |  |  | QUEST-1, phase 3 | [17] |
|  | 1 | no | 81.8 (231) |  |  |  | QUEST-2, phase 3 | [18] |
|  | 1 | no |  | 50.3 (173) |  |  | ATTAIN, phase 3 | [3] |
|  | 1 | no | 83.3 (684) | 49.6 (252) | 1.21×10^-25^ | 9.77×10^-24^ | Combined |  |
|  | 1 | yes |  | 24.6 (61) |  |  | ATTAIN, phase 3 | [3] |
|  | 1 | yes | 58.1 (31) |  |  |  | QUEST-1, phase 3 | [17] |
|  | 1 | yes | 64.7 (17) |  |  |  | QUEST-2, phase 3 | [19] |
|  | 1 | yes | 60.4 (48) | 24.6 (61) | 1.54×10^-4^ | 1.90×10^-4^ | Combined |  |
| Simeprevir + PegIFNα/RBV for 12w and PegIFNα/RBV for 36w | 1 | nd |  | 52 (50) |  |  | ASPIRE, phase 2b | [19] |
|  | 1 | nd | 91.3 (150) |  |  |  | TIGER, phase 3 | [20] |
|  | 1 | nd | 91.3 (150) | 52 (50) | 5.53×10^-10^ | 8.61×10^-9^ | Combined |  |
| Simeprevir + PegIFNα/RBV for 12w and PegIFNα/RBV for 12w or 36w | 4 | nd | 82.9 (35) | 40 (40) | 1.57×10^-4^ | 1.71×10^-4^ | RESTORE, Phase 3 | [21] |
| Sofosbuvir + RBV for 12 weeks | 1 | no | 84 (25) | 10 (10) | 4.26×10^-5^ | 8.72×10^-5^ | Electron, phase 2a | [22] |
| Simeprevir + sofosbuvir for 12w | 1 | no | 100 (4) | 94.1 (17) |  |  | COSMOS, phase 3 | [23] |
|  | 1 | no | 97.4 (115) |  |  |  | OPTIMIST-1, phase 3 | [24] |
|  | 1a | no | 88.4 (69) |  |  |  | HCV-TARGET, phase 3 | [25] |
|  | 1b | no | 97.4 (38) |  |  |  | HCV-TARGET, phase 3 | [25] |
|  | 1 | no | 94.7 (226) | 94.1 (17) | 9.19×10^-1^ | 1.00 | Combined |  |
|  | 1 | yes | 66.7 (3) | 100 (4) |  |  | COSMOS, phase 3 | [23] |
|  | 1 | yes | 88 (50) |  |  |  | OPTIMIST-2, phase 3 | [26] |
|  | 1a | yes | 83.9 (87) |  |  |  | HCV-TARGET, phase 3 | [25] |
|  | 1b | yes | 91.7 (36) |  |  |  | HCV-TARGET, phase 3 | [25] |
|  | 1 | yes | 86.4 (176) | 100 (4) | 4.28×10^-1^ | 1.00 | Combined |  |
| Ledipasvir + sofosbuvir for 12 weeks. | 1 | yes |  | 70 (10) |  |  | Phase 2 | [27] |
|  | 1 | yes | 97 (33) |  |  |  | ION-1, phase 3 | [28] |
|  | 1 | yes | 100 (13) |  |  |  | GS-US-337-0113, phase 3 | [29] |
|  | 1 | yes | 91.7 (527) |  |  |  | Real-world study | [30] |
|  | 1 | yes | 92.1 (573) | 70 (10) | 1.15×10^-2^ | 4.17×10^-2^ | Combined |  |
|  | 1 | nd | 99.1 (214) |  |  |  | ION-1, phase 3 | [28] |
|  | 1 | nd |  | 91.8 (49) |  |  | ION-2, Phase 3 | [31] |
|  | 1 | nd | 99.1 (214) | 91.8 (49) | 2.24×10^-3^ | 1.21×10^-2^ | Combined |  |
| Ledipasvir + sofosbuvir + RBV for 12w | 1 | nd | 97.2 (217) |  |  |  | ION-1, phase 3 | [28] |
|  | 1 | nd |  | 95.7 (46) |  |  | ION-2, Phase 3 | [31] |
|  | 1 | nd | 97.2 (217) | 95.7 (46) | 5.70×10^-1^ | 6.32×10^-1^ | Combined |  |
| Ombitasvir + paritaprevir/ritonavir + dasabuvir for 12w | 1 | no | 88.6 (79) |  |  |  | AVIATOR, phase 2b | [32] |
|  | 1 | no |  | 100 (32) |  |  | PEARL-II, phase 3 | [33] |
|  | 1a | no | 90.2 (205) |  |  |  | PEARL-IV, PEARL-III, phase 3 | [34] |
|  | 1b | no | 99 (209) |  |  |  | PEARL-IV, PEARL-III, phase 3 | [34] |
|  | 1 | no | 100 (7) |  |  |  | RUBY-I, phase 3 | [35] |
|  | 1 | no | 97.6 (83) |  |  |  | MALACHITE-I, phase 3 | [2] |
|  | 1 | no | 97.8 (400) |  |  |  | Real world study | [30] |
|  | 1 | no | 95.7 (983) | 100 (32) | 2.32×10^-1^ | 6.39×10^-1^ | Combined |  |
| Ombitasvir + paritaprevir/ritonavir + dasabuvir + RBV for 12w | 1 | no | 95 (40) | 95.5 (22) |  |  | AVIATOR, phase 2b | [32] |
|  | 1 | no | 97.2 (36) |  |  |  | Phase 2 | [36] |
|  | 1b | no |  | 93.5 (31) |  |  | PEARL-II, phase 3 | [33] |
|  | 1a | no | 97 (100) |  |  |  | PEARL-IV, PEARL-III, phase 3 | [34] |
|  | 1b | no | 99.5 (210) |  |  |  | PEARL-IV, PEARL-III, phase 3 | [34] |
|  | 1a | no | 97.1 (69) |  |  |  | MALACHITE-I | [2] |
|  | 1b | no | 98.8 (84) |  |  |  | MALACHITE-I | [2] |
|  | 1 | no |  | 100 (49) |  |  | MALACHITE-II, phase 3 | [2] |
|  | 1a | no | 95.3 (322) |  |  |  | SAPPHIRE-I, phase 3 | [37] |
|  | 1b | no | 98 (151) |  |  |  | SAPPHIRE-I, phase 3 | [37] |
|  | 1 | no |  | 95.3 (86) |  |  | SAPPHIRE-II, phase 3 | [38] |
|  | 1a | no | 84.6 (13) |  |  |  | RUBY-I, phase 3 | [35] |
|  | 1a | no | 95 (725) |  |  |  | Real world study | [30] |
|  | 1b | no | 98.6 (142) |  |  |  | Real world study | [30] |
|  | 1 | no | 96.4 (1892) | 96.3 (188) | 9.28×10^-1^ | 8.39×10^-1^ | Combined |  |
|  | 1a | yes | 92.2 (64) | 80 (50) |  |  | TURQUOISE-II, phase 3 | [39] |
|  | 1b | yes | 100 (22) | 100 (25) |  |  | TURQUOISE-II, phase 3 | [39] |
|  | 1a | yes | 95.5 (199) |  |  |  | Real world study | [30] |
|  | 1b | yes | 100 (133) |  |  |  | Real world study | [30] |
|  | 1 | yes | 96.7 (418) | 86.7 (75) | 2.16×10^-4^ | 1.22×10^-3^ | Combined |  |
| Grazoprevir + elbasvir for 12w | 1 | yes | 96.6 (29) | 92.9 (14) |  |  | C-WORTHY, phase 2 | [40] |
|  | 1 | yes | 100 (4) |  |  |  | C-SURFER, phase 3 | [41] |
|  | 1a | yes | 93.3 (30) |  |  |  | C-EDGE CO-STAR, phase 3 | [42] |
|  | 1b | yes | 100 (6) |  |  |  | C-EDGE CO-STAR, phase 3 | [42] |
|  | 1a | yes | 94.1 (34) |  |  |  | C-EDGE, phase 3 | [43] |
|  | 1b | yes | 100 (34) |  |  |  | C-EDGE, phase 3 | [43] |
|  | 1 | yes | 96.4 (137) | 92.9 (14) | 5.24×10^-1^ | 4.48×10^-1^ | Combined |  |
|  | 1 | no | 92.9 (85) |  |  |  | C-WORTHY, phase 2 | [44] |
|  | 1 | no |  | 89.5 (19) |  |  | C-WORTHY, phase 2 | [40] |
|  | 1 | no | 92.9 (85) | 89.5 (19) | 6.08×10^-1^ | 6.36×10^-1^ | Combined |  |
|  | 1,4,6 | nd |  | 91.8 (49) |  |  | C-EDGE Experienced Study, phase 3 | [45] |
|  | 1,4,6 | nd | 94.0 (201) |  |  |  | C-EDGE CO-STAR, phase 3 | [42] |
|  | 1,4,6 | nd | 94.6 (316) |  |  |  | C-EDGE, phase 3 | [43] |
|  | 1,4,6 | nd | 94.4 (517) | 91.8 (49) | 4.66×10^-1^ | 5.17×10^-1^ | Combined |  |
| Grazoprevir + elbasvir + RBV for 12w | 1 | yes | 96.9 (32) | 90.9 (11) | 4.18×10^-1^ | 4.51×10^-1^ | C-WORTHY, phase 2 | [40] |
|  | 1 | no | 97.7 (44) |  |  |  | C-WORTHY, phase 2 | [44] |
|  | 1 | no |  | 100 (21) |  |  | C-WORTHY, phase 2 | [40] |
|  | 1 | no | 97.7 (44) | 100 (21) | 4.86×10^-1^ | 1.00 | Combined |  |
| Paritaprevir/rironavir + dasabuvir + RBV for 12w | 1 | no | 94.7 (19) | 47.1 (17) | 1.43×10^-3^ | 2.26×10^-3^ | 2D Phase 2a | [46] |
| Ombitasvir + paritaprevir/ritonavir for 12w | 1b | nd | 95.2 (42) | 90 (40) |  |  | PEARL-I, phase 2b | [47] |
|  | 1b | nd |  | 88.9 (18) |  |  | Japanese 2D, phase 2 | [48] |
|  | 1b | nd | 95.2 (42) | 89.7 (58) | 3.10×10^-1^ | 4.62×10^-1^ | Combined |  |
| Daclatasvir + simeprevir for 12w | 1b | nd | 84.9 (53) | 95 (20) | 2.42×10^-1^ | 4.29×10^-1^ | LEAGUE-1, phase 2 | [49] |
| Daclatasvir + simeprevir + RBV for 12w | 1b | nd | 74.5 (51) | 69.6 (23) | 6.58×10^-1^ | 7.78×10^-1^ | LEAGUE-1, phase 2 | [49] |
| Daclatasvir + asunaprevir for 12w | 1 | no | 89.5 (171) | 79.6 (142) | 1.47×10^-2^ | 1.72×10^-2^ | HALLMARK-DUAL, phase 3 | [50] |
|  | 1 | yes | 90.6 (32) | 87.3 (63) | 6.32×10^-1^ | 7.45×10^-1^ | HALLMARK-DUAL, phase 3 | [50] |
| Sofosbuvir + radalbuvir + RBV | 1 | no | 92 (25) | 100 (10) | 3.57×10^-1^ | 1.00 | Phase 2 | [27] |
| Daclatasvir + asunaprevir + beclabuvir for 12 w | 1 | no | 92.0 (312) | 88.0 (25) | 4.87×10^-1^ | 4.50×10^-1^ | UNITY-1, phase 3 | [51] |
| Daclatasvir + asunaprevir + beclabuvir ± RBV for 24 w | 1 | yes | 95.5 (112) | 97.1 (35) | 6.75×10^-1^ | 1.00 | UNITY-2, phase 3 | [52] |

**S1 Table References**

1. Vertex. Incivek highlights of prescribing information. <http://pi.vrtx.com/files/uspi_telaprevir.pdf>. 2013.

2. Dore GJ, Conway B, Luo Y, Janczewska E, Knysz B, Liu Y, et al. Efficacy and safety of ombitasvir/paritaprevir/r and dasabuvir compared to IFN-containing regimens in genotype 1 HCV patients: The MALACHITE-I/II trials. J Hepatol. 2016;64:19-28.

3. Reddy KR, Zeuzem S, Zoulim F, Weiland O, Horban A, Stanciu C, et al. Simeprevir versus telaprevir with peginterferon and ribavirin in previous null or partial responders with chronic hepatitis C virus genotype 1 infection (ATTAIN): a randomised, double-blind, non-inferiority phase 3 trial. Lancet Infect Dis. 2015;15:27-35.

4. Kumada H, Suzuki F, Suzuki Y, Toyota J, Karino Y, Chayama K, et al. Randomized comparison of daclatasvir + asunaprevir versus telaprevir + peginterferon/ribavirin in Japanese hepatitis C virus patients. J Gastroenterol Hepatol. 2016;31:14-22.

5. Buti M, Agarwal K, Horsmans Y, Sievert W, Janczewska E, Zeuzem S, et al. Telaprevir twice daily is noninferior to telaprevir every 8 hours for patients with chronic hepatitis C. Gastroenterology. 2014;146:744-753 e743.

6. Sterling RK, Kuo A, Rustgi VK, Sulkowski MS, Stewart TG, Fenkel JM, et al. Virological outcomes and treatment algorithms utilisation in observational study of patients with chronic hepatitis C treated with boceprevir or telaprevir. Aliment Pharmacol Ther. 2015;41:671-685.

7. Kwo PY, Lawitz EJ, McCone J, Schiff ER, Vierling JM, Pound D, et al. Efficacy of boceprevir, an NS3 protease inhibitor, in combination with peginterferon alfa-2b and ribavirin in treatment-naive patients with genotype 1 hepatitis C infection (SPRINT-1): an open-label, randomised, multicentre phase 2 trial. Lancet. 2010;376:705-716.

8. Hezode C, Fontaine H, Dorival C, Zoulim F, Larrey D, Canva V, et al. Effectiveness of telaprevir or boceprevir in treatment-experienced patients with HCV genotype 1 infection and cirrhosis. Gastroenterology. 2014;147:132-142 e134.

9. Poordad F, McCone J, Jr., Bacon BR, Bruno S, Manns MP, Sulkowski MS, et al. Boceprevir for untreated chronic HCV genotype 1 infection. N Engl J Med. 2011;364:1195-1206.

10. Vierling JM, Davis M, Flamm S, Gordon SC, Lawitz E, Yoshida EM, et al. Boceprevir for chronic HCV genotype 1 infection in patients with prior treatment failure to peginterferon/ribavirin, including prior null response. J Hepatol. 2014;60:748-756.

11. Poordad F, Lawitz E, Reddy KR, Afdhal NH, Hezode C, Zeuzem S, et al. Effects of ribavirin dose reduction vs erythropoietin for boceprevir-related anemia in patients with chronic hepatitis C virus genotype 1 infection--a randomized trial. Gastroenterology. 2013;145:1035-1044 e1035.

12. Flamm SL, Lawitz E, Jacobson I, Bourliere M, Hezode C, Vierling JM, et al. Boceprevir with peginterferon alfa-2a-ribavirin is effective for previously treated chronic hepatitis C genotype 1 infection. Clin Gastroenterol Hepatol. 2013;11:81-87 e84; quiz e85.

13. Fried MW, Buti M, Dore GJ, Flisiak R, Ferenci P, Jacobson I, et al. Once-daily simeprevir (TMC435) with pegylated interferon and ribavirin in treatment-naive genotype 1 hepatitis C: the randomized PILLAR study. Hepatology. 2013;58:1918-1929.

14. Hayashi N, Izumi N, Kumada H, Okanoue T, Tsubouchi H, Yatsuhashi H, et al. Simeprevir with peginterferon/ribavirin for treatment-naive hepatitis C genotype 1 patients in Japan: CONCERTO-1, a phase III trial. J Hepatol. 2014;61:219-227.

15. Izumi N, Hayashi N, Kumada H, Okanoue T, Tsubouchi H, Yatsuhashi H, et al. Once-daily simeprevir with peginterferon and ribavirin for treatment-experienced HCV genotype 1-infected patients in Japan: the CONCERTO-2 and CONCERTO-3 studies. J Gastroenterol. 2014;49:941-953.

16. Kumada H, Hayashi N, Izumi N, Okanoue T, Tsubouchi H, Yatsuhashi H, et al. Simeprevir (TMC435) once daily with peginterferon-alpha-2b and ribavirin in patients with genotype 1 hepatitis C virus infection: The CONCERTO-4 study. Hepatol Res. 2015;45:501-513.

17. Jacobson IM, Dore GJ, Foster GR, Fried MW, Radu M, Rafalsky VV, et al. Simeprevir with pegylated interferon alfa 2a plus ribavirin in treatment-naive patients with chronic hepatitis C virus genotype 1 infection (QUEST-1): a phase 3, randomised, double-blind, placebo-controlled trial. Lancet. 2014;384:403-413.

18. Manns M, Marcellin P, Poordad F, de Araujo ES, Buti M, Horsmans Y, et al. Simeprevir with pegylated interferon alfa 2a or 2b plus ribavirin in treatment-naive patients with chronic hepatitis C virus genotype 1 infection (QUEST-2): a randomised, double-blind, placebo-controlled phase 3 trial. Lancet. 2014;384:414-426.

19. Zeuzem S, Berg T, Gane E, Ferenci P, Foster GR, Fried MW, et al. Simeprevir increases rate of sustained virologic response among treatment-experienced patients with HCV genotype-1 infection: a phase IIb trial. Gastroenterology. 2014;146:430-441.

20. Wei L, Han T, Yang D, Heo J, Shang J, Cheng J, et al. Simeprevir plus peginterferon/ribavirin for HCV genotype 1-infected treatment-naive patients in China and South Korea. J Gastroenterol Hepatol. 2016;31:912-920.

21. Moreno C, Hezode C, Marcellin P, Bourgeois S, Francque S, Samuel D, et al. Efficacy and safety of simeprevir with PegIFN/ribavirin in naive or experienced patients infected with chronic HCV genotype 4. J Hepatol. 2015;62:1047-1055.

22. Gane EJ, Stedman CA, Hyland RH, Ding X, Svarovskaia E, Symonds WT, et al. Nucleotide polymerase inhibitor sofosbuvir plus ribavirin for hepatitis C. N Engl J Med. 2013;368:34-44.

23. Lawitz E, Sulkowski MS, Ghalib R, Rodriguez-Torres M, Younossi ZM, Corregidor A, et al. Simeprevir plus sofosbuvir, with or without ribavirin, to treat chronic infection with hepatitis C virus genotype 1 in non-responders to pegylated interferon and ribavirin and treatment-naive patients: the COSMOS randomised study. Lancet. 2014;384:1756-1765.

24. Kwo P, Gitlin N, Nahass R, Bernstein D, Etzkorn K, Rojter S, et al. Simeprevir plus sofosbuvir (12 and 8 weeks) in hepatitis C virus genotype 1-infected patients without cirrhosis: OPTIMIST-1, a phase 3, randomized study. Hepatology. 2016;64:370-380.

25. Sulkowski MS, Vargas HE, Di Bisceglie AM, Kuo A, Reddy KR, Lim JK, et al. Effectiveness of simeprevir plus sofosbuvir, with or without ribavirin, in real-world patients with HCV genotype 1 infection. Gastroenterology. 2016;150:419-429.

26. Lawitz E, Matusow G, DeJesus E, Yoshida EM, Felizarta F, Ghalib R, et al. Simeprevir plus sofosbuvir in patients with chronic hepatitis C virus genotype 1 infection and cirrhosis: A phase 3 study (OPTIMIST-2). Hepatology. 2016;64:360-369.

27. Gane EJ, Stedman CA, Hyland RH, Ding X, Svarovskaia E, Subramanian GM, et al. Efficacy of nucleotide polymerase inhibitor sofosbuvir plus the NS5A inhibitor ledipasvir or the NS5B non-nucleoside inhibitor GS-9669 against HCV genotype 1 infection. Gastroenterology. 2014;146:736-743 e731.

28. Afdhal N, Zeuzem S, Kwo P, Chojkier M, Gitlin N, Puoti M, et al. Ledipasvir and sofosbuvir for untreated HCV genotype 1 infection. N Engl J Med. 2014;370:1889-1898.

29. Mizokami M, Yokosuka O, Takehara T, Sakamoto N, Korenaga M, Mochizuki H, et al. Ledipasvir and sofosbuvir fixed-dose combination with and without ribavirin for 12 weeks in treatment-naive and previously treated Japanese patients with genotype 1 hepatitis C: an open-label, randomised, phase 3 trial. Lancet Infect Dis. 2015;15:645-653.

30. Ioannou GN, Beste LA, Chang MF, Green PK, Lowy E, Tsui JI, et al. Effectiveness of sofosbuvir, ledipasvir/sofosbuvir, or paritaprevir/ritonavir/ombitasvir and dasabuvir regimens for treatment of patients with hepatitis C in the veterans affairs national health care system. Gastroenterology. 2016;151:457-471 e455.

31. Afdhal N, Reddy KR, Nelson DR, Lawitz E, Gordon SC, Schiff E, et al. Ledipasvir and sofosbuvir for previously treated HCV genotype 1 infection. N Engl J Med. 2014;370:1483-1493.

32. Kowdley KV, Lawitz E, Poordad F, Cohen DE, Nelson DR, Zeuzem S, et al. Phase 2b trial of interferon-free therapy for hepatitis C virus genotype 1. N Engl J Med. 2014;370:222-232.

33. Andreone P, Colombo MG, Enejosa JV, Koksal I, Ferenci P, Maieron A, et al. ABT-450, ritonavir, ombitasvir, and dasabuvir achieves 97% and 100% sustained virologic response with or without ribavirin in treatment-experienced patients with HCV genotype 1b infection. Gastroenterology. 2014;147:359-365 e351.

34. Ferenci P, Bernstein D, Lalezari J, Cohen D, Luo Y, Cooper C, et al. ABT-450/r-ombitasvir and dasabuvir with or without ribavirin for HCV. N Engl J Med. 2014;370:1983-1992.

35. Pockros PJ, Reddy KR, Mantry PS, Cohen E, Bennett M, Sulkowski MS, et al. Efficacy of direct-acting antiviral combination for patients with hepatitis C virus genotype 1 infection and severe renal impairment or end-stage renal disease. Gastroenterology. 2016;150:1590-1598.

36. Lalezari J, Sullivan JG, Varunok P, Galen E, Kowdley KV, Rustgi V, et al. Ombitasvir/paritaprevir/r and dasabuvir plus ribavirin in HCV genotype 1-infected patients on methadone or buprenorphine. J Hepatol. 2015;63:364-369.

37. Feld JJ, Kowdley KV, Coakley E, Sigal S, Nelson DR, Crawford D, et al. Treatment of HCV with ABT-450/r-ombitasvir and dasabuvir with ribavirin. N Engl J Med. 2014;370:1594-1603.

38. Zeuzem S, Jacobson IM, Baykal T, Marinho RT, Poordad F, Bourliere M, et al. Retreatment of HCV with ABT-450/r-ombitasvir and dasabuvir with ribavirin. N Engl J Med. 2014;370:1604-1614.

39. Poordad F, Hezode C, Trinh R, Kowdley KV, Zeuzem S, Agarwal K, et al. ABT-450/r-ombitasvir and dasabuvir with ribavirin for hepatitis C with cirrhosis. N Engl J Med. 2014;370:1973-1982.

40. Lawitz E, Gane E, Pearlman B, Tam E, Ghesquiere W, Guyader D, et al. Efficacy and safety of 12 weeks versus 18 weeks of treatment with grazoprevir (MK-5172) and elbasvir (MK-8742) with or without ribavirin for hepatitis C virus genotype 1 infection in previously untreated patients with cirrhosis and patients with previous null response with or without cirrhosis (C-WORTHY): a randomised, open-label phase 2 trial. Lancet. 2015;385:1075-1086.

41. Roth D, Nelson DR, Bruchfeld A, Liapakis A, Silva M, Monsour H, Jr., et al. Grazoprevir plus elbasvir in treatment-naive and treatment-experienced patients with hepatitis C virus genotype 1 infection and stage 4-5 chronic kidney disease (the C-SURFER study): a combination phase 3 study. Lancet. 2015;386:1537-1545.

42. Dore GJ, Altice F, Litwin AH, Dalgard O, Gane EJ, Shibolet O, et al. Elbasvir-Grazoprevir to treat hepatitis C virus infection in persons receiving opioid agonist therapy: A randomized trial. Ann Intern Med. 2016;165:625-634.

43. Zeuzem S, Ghalib R, Reddy KR, Pockros PJ, Ben Ari Z, Zhao Y, et al. Grazoprevir-elbasvir combination therapy for treatment-naive cirrhotic and noncirrhotic patients with chronic hepatitis C virus genotype 1, 4, or 6 infection: a randomized trial. Ann Intern Med. 2015;163:1-13.

44. Sulkowski M, Hezode C, Gerstoft J, Vierling JM, Mallolas J, Pol S, et al. Efficacy and safety of 8 weeks versus 12 weeks of treatment with grazoprevir (MK-5172) and elbasvir (MK-8742) with or without ribavirin in patients with hepatitis C virus genotype 1 mono-infection and HIV/hepatitis C virus co-infection (C-WORTHY): a randomised, open-label phase 2 trial. Lancet. 2015;385:1087-1097.

45. Kwo P, Gane EJ, Peng CY, Pearlman B, Vierling JM, Serfaty L, et al. Effectiveness of elbasvir and grazoprevir combination, with or without ribavirin, for treatment-experienced patients with chronic hepatitis C infection. Gastroenterology. 2017;152:164-175 e164.

46. Poordad F, Lawitz E, Kowdley KV, Cohen DE, Podsadecki T, Siggelkow S, et al. Exploratory study of oral combination antiviral therapy for hepatitis C. N Engl J Med. 2013;368:45-53.

47. Lawitz E, Makara M, Akarca US, Thuluvath PJ, Preotescu LL, Varunok P, et al. Efficacy and safety of ombitasvir, paritaprevir, and ritonavir in an open-label study of patients with genotype 1b chronic hepatitis C virus infection with and without cirrhosis. Gastroenterology. 2015;149:971-980 e971.

48. Chayama K, Notsumata K, Kurosaki M, Sato K, Rodrigues L, Jr., Setze C, et al. Randomized trial of interferon- and ribavirin-free ombitasvir/paritaprevir/ritonavir in treatment-experienced hepatitis C virus-infected patients. Hepatology. 2015;61:1523-1532.

49. Zeuzem S, Hezode C, Bronowicki JP, Loustaud-Ratti V, Gea F, Buti M, et al. Daclatasvir plus simeprevir with or without ribavirin for the treatment of chronic hepatitis C virus genotype 1 infection. J Hepatol. 2016;64:292-300.

50. Manns M, Pol S, Jacobson IM, Marcellin P, Gordon SC, Peng CY, et al. All-oral daclatasvir plus asunaprevir for hepatitis C virus genotype 1b: a multinational, phase 3, multicohort study. Lancet. 2014;384:1597-1605.

51. Poordad F, Sievert W, Mollison L, Bennett M, Tse E, Brau N, et al. Fixed-dose combination therapy with daclatasvir, asunaprevir, and beclabuvir for noncirrhotic patients with HCV genotype 1 infection. J Amer Med Assoc. 2015;313:1728-1735.

52. Muir AJ, Poordad F, Lalezari J, Everson G, Dore GJ, Herring R, et al. Daclatasvir in combination with asunaprevir and beclabuvir for hepatitis C virus genotype 1 infection with compensated cirrhosis. J Amer Med Assoc. 2015;313:1736-1744.
