# Supplementary material for: Modelling how responsiveness to interferon improves interferon-free treatment of hepatitis C virus infection
Source: PLoS Comput Biol. 2018 Jul 12;14(7):e1006335. doi: 10.1371/journal.pcbi.1006335 (PMC6057683; doi:10.1371/journal.pcbi.1006335)
Supplement: S2 Table — The datasets in S1 Table that consider patients with liver cirrhosis alone are summarized. (DOCX) [file pcbi.1006335.s005.docx]

**S2 Table. Response to DAA-based treatments in patients with liver cirrhosis.** The datasets in S1 Table that consider patients with liver cirrhosis alone are summarized.

|  | **Regimen** | **Genotype** | **% SVR (N)** | | **P-value** | | **Ref.** |
| --- | --- | --- | --- | --- | --- | --- | --- |
|  |  |  | **Naïve** | **Null** | **χ^2^** | **Fisher** |  |
| IFN based | Telaprevir + PegIFNα/RBV | 1 | 45.4 (291) | 26.6 (79) | 6.70×10^-7^ | 1.46×10^-6^ | [1-4] |
|  | Boceprevir + PegIFNα/RBV | 1 | 44.3 (140) | 0 (10) | 5.03×10^-5^ | 9.24×10^-5^ | [3-7] |
|  | Simeprevir + PegIFNα/RBV | 1 | 60.4 (48) | 24.6 (61) | 1.21×10^-25^ | 9.77×10^-24^ | [1, 8-10] |
| IFN free | Simeprevir + sofosbuvir | 1 | 86.4 (176) | 100 (4) | 4.26×10^-5^ | 8.72×10^-5^ | [11-13] |
|  | Ledipasvir + sofosbuvir | 1 | 92.1 (573) | 70 (10) | 9.19×10^-1^ | 1.00 | [14-17] |
|  | Ombitasvir + paritaprevir/ritonavir + dasabuvir + RBV | 1 | 96.7 (418) | 86.7 (75) | 2.32×10^-1^ | 6.39×10^-1^ | [17, 18] |
|  | Grazoprevir + elbasvir | 1 | 96.4 (137) | 92.9 (14) | 9.28×10^-1^ | 8.39×10^-1^ | [19-22] |
|  | Grazoprevir + elbasvir + RBV | 1 | 96.9 (32) | 90.9 (11) | 1.43×10^-3^ | 2.26×10^-3^ | [19] |
|  | Daclatasvir + asunaprevir | 1 | 90.6 (32) | 87.3 (63) | 1.47×10^-2^ | 1.72×10^-2^ | [23] |
|  | Daclatasvir + asunaprevir + beclabuvir ± RBV | 1 | 95.5 (112) | 97.1 (35) | 6.75×10^-1^ | 1.00 | [24] |

**S2 Table References**

1. Reddy KR, Zeuzem S, Zoulim F, Weiland O, Horban A, Stanciu C, et al. Simeprevir versus telaprevir with peginterferon and ribavirin in previous null or partial responders with chronic hepatitis C virus genotype 1 infection (ATTAIN): a randomised, double-blind, non-inferiority phase 3 trial. Lancet Infect Dis. 2015;15:27-35.

2. Buti M, Agarwal K, Horsmans Y, Sievert W, Janczewska E, Zeuzem S, et al. Telaprevir twice daily is noninferior to telaprevir every 8 hours for patients with chronic hepatitis C. Gastroenterology. 2014;146:744-753 e743.

3. Sterling RK, Kuo A, Rustgi VK, Sulkowski MS, Stewart TG, Fenkel JM, et al. Virological outcomes and treatment algorithms utilisation in observational study of patients with chronic hepatitis C treated with boceprevir or telaprevir. Aliment Pharmacol Ther. 2015;41:671-685.

4. Zeuzem S, Berg T, Gane E, Ferenci P, Foster GR, Fried MW, et al. Simeprevir increases rate of sustained virologic response among treatment-experienced patients with HCV genotype-1 infection: a phase IIb trial. Gastroenterology. 2014;146:430-441.

5. Kwo PY, Lawitz EJ, McCone J, Schiff ER, Vierling JM, Pound D, et al. Efficacy of boceprevir, an NS3 protease inhibitor, in combination with peginterferon alfa-2b and ribavirin in treatment-naive patients with genotype 1 hepatitis C infection (SPRINT-1): an open-label, randomised, multicentre phase 2 trial. Lancet. 2010;376:705-716.

6. Poordad F, McCone J, Jr., Bacon BR, Bruno S, Manns MP, Sulkowski MS, et al. Boceprevir for untreated chronic HCV genotype 1 infection. N Engl J Med. 2011;364:1195-1206.

7. Poordad F, Lawitz E, Reddy KR, Afdhal NH, Hezode C, Zeuzem S, et al. Effects of ribavirin dose reduction vs erythropoietin for boceprevir-related anemia in patients with chronic hepatitis C virus genotype 1 infection--a randomized trial. Gastroenterology. 2013;145:1035-1044 e1035.

8. Kumada H, Hayashi N, Izumi N, Okanoue T, Tsubouchi H, Yatsuhashi H, et al. Simeprevir (TMC435) once daily with peginterferon-alpha-2b and ribavirin in patients with genotype 1 hepatitis C virus infection: The CONCERTO-4 study. Hepatol Res. 2015;45:501-513.

9. Jacobson IM, Dore GJ, Foster GR, Fried MW, Radu M, Rafalsky VV, et al. Simeprevir with pegylated interferon alfa 2a plus ribavirin in treatment-naive patients with chronic hepatitis C virus genotype 1 infection (QUEST-1): a phase 3, randomised, double-blind, placebo-controlled trial. Lancet. 2014;384:403-413.

10. Manns M, Marcellin P, Poordad F, de Araujo ES, Buti M, Horsmans Y, et al. Simeprevir with pegylated interferon alfa 2a or 2b plus ribavirin in treatment-naive patients with chronic hepatitis C virus genotype 1 infection (QUEST-2): a randomised, double-blind, placebo-controlled phase 3 trial. Lancet. 2014;384:414-426.

11. Lawitz E, Sulkowski MS, Ghalib R, Rodriguez-Torres M, Younossi ZM, Corregidor A, et al. Simeprevir plus sofosbuvir, with or without ribavirin, to treat chronic infection with hepatitis C virus genotype 1 in non-responders to pegylated interferon and ribavirin and treatment-naive patients: the COSMOS randomised study. Lancet. 2014;384:1756-1765.

12. Sulkowski MS, Vargas HE, Di Bisceglie AM, Kuo A, Reddy KR, Lim JK, et al. Effectiveness of simeprevir plus sofosbuvir, with or without ribavirin, in real-world patients with HCV genotype 1 infection. Gastroenterology. 2016;150:419-429.

13. Lawitz E, Matusow G, DeJesus E, Yoshida EM, Felizarta F, Ghalib R, et al. Simeprevir plus sofosbuvir in patients with chronic hepatitis C virus genotype 1 infection and cirrhosis: A phase 3 study (OPTIMIST-2). Hepatology. 2016;64:360-369.

14. Gane EJ, Stedman CA, Hyland RH, Ding X, Svarovskaia E, Subramanian GM, et al. Efficacy of nucleotide polymerase inhibitor sofosbuvir plus the NS5A inhibitor ledipasvir or the NS5B non-nucleoside inhibitor GS-9669 against HCV genotype 1 infection. Gastroenterology. 2014;146:736-743 e731.

15. Afdhal N, Zeuzem S, Kwo P, Chojkier M, Gitlin N, Puoti M, et al. Ledipasvir and sofosbuvir for untreated HCV genotype 1 infection. N Engl J Med. 2014;370:1889-1898.

16. Mizokami M, Yokosuka O, Takehara T, Sakamoto N, Korenaga M, Mochizuki H, et al. Ledipasvir and sofosbuvir fixed-dose combination with and without ribavirin for 12 weeks in treatment-naive and previously treated Japanese patients with genotype 1 hepatitis C: an open-label, randomised, phase 3 trial. Lancet Infect Dis. 2015;15:645-653.

17. Ioannou GN, Beste LA, Chang MF, Green PK, Lowy E, Tsui JI, et al. Effectiveness of sofosbuvir, ledipasvir/sofosbuvir, or paritaprevir/ritonavir/ombitasvir and dasabuvir regimens for treatment of patients with hepatitis C in the veterans affairs national health care system. Gastroenterology. 2016;151:457-471 e455.

18. Poordad F, Hezode C, Trinh R, Kowdley KV, Zeuzem S, Agarwal K, et al. ABT-450/r-ombitasvir and dasabuvir with ribavirin for hepatitis C with cirrhosis. N Engl J Med. 2014;370:1973-1982.

19. Lawitz E, Gane E, Pearlman B, Tam E, Ghesquiere W, Guyader D, et al. Efficacy and safety of 12 weeks versus 18 weeks of treatment with grazoprevir (MK-5172) and elbasvir (MK-8742) with or without ribavirin for hepatitis C virus genotype 1 infection in previously untreated patients with cirrhosis and patients with previous null response with or without cirrhosis (C-WORTHY): a randomised, open-label phase 2 trial. Lancet. 2015;385:1075-1086.

20. Roth D, Nelson DR, Bruchfeld A, Liapakis A, Silva M, Monsour H, Jr., et al. Grazoprevir plus elbasvir in treatment-naive and treatment-experienced patients with hepatitis C virus genotype 1 infection and stage 4-5 chronic kidney disease (the C-SURFER study): a combination phase 3 study. Lancet. 2015;386:1537-1545.

21. Dore GJ, Altice F, Litwin AH, Dalgard O, Gane EJ, Shibolet O, et al. Elbasvir-grazoprevir to treat hepatitis c virus infection in persons receiving opioid agonist therapy: A randomized trial. Ann Intern Med. 2016;165:625-634.

22. Zeuzem S, Ghalib R, Reddy KR, Pockros PJ, Ben Ari Z, Zhao Y, et al. Grazoprevir-elbasvir combination therapy for treatment-naive cirrhotic and noncirrhotic patients with chronic hepatitis C virus genotype 1, 4, or 6 infection: a randomized trial. Ann Intern Med. 2015;163:1-13.

23. Manns M, Pol S, Jacobson IM, Marcellin P, Gordon SC, Peng CY, et al. All-oral daclatasvir plus asunaprevir for hepatitis C virus genotype 1b: a multinational, phase 3, multicohort study. Lancet. 2014;384:1597-1605.

24. Muir AJ, Poordad F, Lalezari J, Everson G, Dore GJ, Herring R, et al. Daclatasvir in combination with asunaprevir and beclabuvir for hepatitis C virus genotype 1 infection with compensated cirrhosis. J Amer Med Assoc. 2015;313:1736-1744.
