# Supplementary material for: Modelling how responsiveness to interferon improves interferon-free treatment of hepatitis C virus infection
Source: PLoS Comput Biol. 2018 Jul 12;14(7):e1006335. doi: 10.1371/journal.pcbi.1006335 (PMC6057683; doi:10.1371/journal.pcbi.1006335)
Supplement: S3 Table — The datasets in S1 Table that consider patients without liver cirrhosis alone are summarized. (DOCX) [file pcbi.1006335.s006.docx]

**S3 Table. Response to DAA-based treatments in patients without liver cirrhosis.** The datasets in S1 Table that consider patients without liver cirrhosis alone are summarized.

|  | **Regimen** | **Genotype** | **% SVR (N)** | | **P-value** | | **Ref.** |
| --- | --- | --- | --- | --- | --- | --- | --- |
|  |  |  | **Naïve** | **Null** | **χ^2^** | **Fisher** |  |
| IFN based | Telaprevir + PegIFNα/RBV | 1 | 68.7 (941) | 50.7 (213) | 2.65×10^-3^ | 2.94×10^-3^ | [1-5] |
|  | Boceprevir + PegIFNα/RBV | 1 | 65.4 (1179) | 43.5 (85) | 6.01×10^-3^ | 5.48×10^-3^ | [5-9] |
|  | Simeprevir + PegIFNα/RBV | 1 | 83.3 (684) | 49.6 (252) | 1.54×10^-4^ | 1.90×10^-4^ | [2, 10-15] |
| IFN free | Sofosbuvir + RBV | 1 | 84 (25) | 10 (10) | 4.28×10^-1^ | 1.00 | [16] |
|  | Simeprevir + sofosbuvir | 1 | 94.7 (226) | 94.1 (17) | 1.15×10^-2^ | 4.17×10^-2^ | [17-19] |
|  | Ombitasvir + paritaprevir/ritonavir + dasabuvir | 1 | 95.7 (983) | 100 (32) | 2.16×10^-4^ | 1.22×10^-3^ | [1, 20-24] |
|  | Ombitasvir + paritaprevir/ritonavir + dasabuvir + RBV | 1 | 96.4 (1892) | 96.3 (188) | 5.24×10^-1^ | 4.48×10^-1^ | [1, 20-27] |
|  | Grazoprevir + elbasvir | 1 | 92.9 (85) | 89.5 (19) | 6.08×10^-1^ | 6.36×10^-1^ | [28, 29] |
|  | Grazoprevir + elbasvir + RBV | 1 | 97.7 (44) | 100 (21) | 4.86×10^-1^ | 1.00 | [28, 29] |
|  | Paritaprevir/ritonavir + dasabuvir + RBV | 1 | 94.7 (19) | 47.1 (17) | 4.18×10^-1^ | 4.51×10^-1^ | [30] |
|  | Daclatasvir + asunaprevir | 1 | 89.5 (171) | 79.6 (142) | 6.32×10^-1^ | 7.45×10^-1^ | [31] |
|  | Sofosbuvir + radalbuvir + RBV | 1 | 92 (25) | 100 (10) | 2.65×10^-3^ | 2.94×10^-3^ | [32] |
|  | Daclatasvir + asunaprevir + beclabuvir | 1 | 92.0 (312) | 88.0 (25) | 4.87×10^-1^ | 4.50×10^-1^ | [33] |

**S3 Table References**

1. Dore GJ, Conway B, Luo Y, Janczewska E, Knysz B, Liu Y, et al. Efficacy and safety of ombitasvir/paritaprevir/r and dasabuvir compared to IFN-containing regimens in genotype 1 HCV patients: The MALACHITE-I/II trials. J Hepatol. 2016;64:19-28.

2. Reddy KR, Zeuzem S, Zoulim F, Weiland O, Horban A, Stanciu C, et al. Simeprevir versus telaprevir with peginterferon and ribavirin in previous null or partial responders with chronic hepatitis C virus genotype 1 infection (ATTAIN): a randomised, double-blind, non-inferiority phase 3 trial. Lancet Infect Dis. 2015;15:27-35.

3. Kumada H, Suzuki F, Suzuki Y, Toyota J, Karino Y, Chayama K, et al. Randomized comparison of daclatasvir + asunaprevir versus telaprevir + peginterferon/ribavirin in Japanese hepatitis C virus patients. J Gastroenterol Hepatol. 2016;31:14-22.

4. Buti M, Agarwal K, Horsmans Y, Sievert W, Janczewska E, Zeuzem S, et al. Telaprevir twice daily is noninferior to telaprevir every 8 hours for patients with chronic hepatitis C. Gastroenterology. 2014;146:744-753 e743.

5. Sterling RK, Kuo A, Rustgi VK, Sulkowski MS, Stewart TG, Fenkel JM, et al. Virological outcomes and treatment algorithms utilisation in observational study of patients with chronic hepatitis C treated with boceprevir or telaprevir. Aliment Pharmacol Ther. 2015;41:671-685.

6. Kwo PY, Lawitz EJ, McCone J, Schiff ER, Vierling JM, Pound D, et al. Efficacy of boceprevir, an NS3 protease inhibitor, in combination with peginterferon alfa-2b and ribavirin in treatment-naive patients with genotype 1 hepatitis C infection (SPRINT-1): an open-label, randomised, multicentre phase 2 trial. Lancet. 2010;376:705-716.

7. Poordad F, McCone J, Jr., Bacon BR, Bruno S, Manns MP, Sulkowski MS, et al. Boceprevir for untreated chronic HCV genotype 1 infection. N Engl J Med. 2011;364:1195-1206.

8. Vierling JM, Davis M, Flamm S, Gordon SC, Lawitz E, Yoshida EM, et al. Boceprevir for chronic HCV genotype 1 infection in patients with prior treatment failure to peginterferon/ribavirin, including prior null response. J Hepatol. 2014;60:748-756.

9. Poordad F, Lawitz E, Reddy KR, Afdhal NH, Hezode C, Zeuzem S, et al. Effects of ribavirin dose reduction vs erythropoietin for boceprevir-related anemia in patients with chronic hepatitis C virus genotype 1 infection--a randomized trial. Gastroenterology. 2013;145:1035-1044 e1035.

10. Fried MW, Buti M, Dore GJ, Flisiak R, Ferenci P, Jacobson I, et al. Once-daily simeprevir (TMC435) with pegylated interferon and ribavirin in treatment-naive genotype 1 hepatitis C: the randomized PILLAR study. Hepatology. 2013;58:1918-1929.

11. Hayashi N, Izumi N, Kumada H, Okanoue T, Tsubouchi H, Yatsuhashi H, et al. Simeprevir with peginterferon/ribavirin for treatment-naive hepatitis C genotype 1 patients in Japan: CONCERTO-1, a phase III trial. J Hepatol. 2014;61:219-227.

12. Izumi N, Hayashi N, Kumada H, Okanoue T, Tsubouchi H, Yatsuhashi H, et al. Once-daily simeprevir with peginterferon and ribavirin for treatment-experienced HCV genotype 1-infected patients in Japan: the CONCERTO-2 and CONCERTO-3 studies. J Gastroenterol. 2014;49:941-953.

13. Kumada H, Hayashi N, Izumi N, Okanoue T, Tsubouchi H, Yatsuhashi H, et al. Simeprevir (TMC435) once daily with peginterferon-alpha-2b and ribavirin in patients with genotype 1 hepatitis C virus infection: The CONCERTO-4 study. Hepatol Res. 2015;45:501-513.

14. Jacobson IM, Dore GJ, Foster GR, Fried MW, Radu M, Rafalsky VV, et al. Simeprevir with pegylated interferon alfa 2a plus ribavirin in treatment-naive patients with chronic hepatitis C virus genotype 1 infection (QUEST-1): a phase 3, randomised, double-blind, placebo-controlled trial. Lancet. 2014;384:403-413.

15. Manns M, Marcellin P, Poordad F, de Araujo ES, Buti M, Horsmans Y, et al. Simeprevir with pegylated interferon alfa 2a or 2b plus ribavirin in treatment-naive patients with chronic hepatitis C virus genotype 1 infection (QUEST-2): a randomised, double-blind, placebo-controlled phase 3 trial. Lancet. 2014;384:414-426.

16. Gane EJ, Stedman CA, Hyland RH, Ding X, Svarovskaia E, Symonds WT, et al. Nucleotide polymerase inhibitor sofosbuvir plus ribavirin for hepatitis C. N Engl J Med. 2013;368:34-44.

17. Lawitz E, Sulkowski MS, Ghalib R, Rodriguez-Torres M, Younossi ZM, Corregidor A, et al. Simeprevir plus sofosbuvir, with or without ribavirin, to treat chronic infection with hepatitis C virus genotype 1 in non-responders to pegylated interferon and ribavirin and treatment-naive patients: the COSMOS randomised study. Lancet. 2014;384:1756-1765.

18. Kwo P, Gitlin N, Nahass R, Bernstein D, Etzkorn K, Rojter S, et al. Simeprevir plus sofosbuvir (12 and 8 weeks) in hepatitis C virus genotype 1-infected patients without cirrhosis: OPTIMIST-1, a phase 3, randomized study. Hepatology. 2016;64:370-380.

19. Sulkowski MS, Vargas HE, Di Bisceglie AM, Kuo A, Reddy KR, Lim JK, et al. Effectiveness of simeprevir plus sofosbuvir, with or without ribavirin, in real-world patients with HCV genotype 1 infection. Gastroenterology. 2016;150:419-429.

20. Ioannou GN, Beste LA, Chang MF, Green PK, Lowy E, Tsui JI, et al. Effectiveness of sofosbuvir, ledipasvir/sofosbuvir, or paritaprevir/ritonavir/ombitasvir and dasabuvir regimens for treatment of patients with hepatitis C in the veterans affairs national health care system. Gastroenterology. 2016;151:457-471 e455.

21. Kowdley KV, Lawitz E, Poordad F, Cohen DE, Nelson DR, Zeuzem S, et al. Phase 2b trial of interferon-free therapy for hepatitis C virus genotype 1. N Engl J Med. 2014;370:222-232.

22. Andreone P, Colombo MG, Enejosa JV, Koksal I, Ferenci P, Maieron A, et al. ABT-450, ritonavir, ombitasvir, and dasabuvir achieves 97% and 100% sustained virologic response with or without ribavirin in treatment-experienced patients with HCV genotype 1b infection. Gastroenterology. 2014;147:359-365 e351.

23. Ferenci P, Bernstein D, Lalezari J, Cohen D, Luo Y, Cooper C, et al. ABT-450/r-ombitasvir and dasabuvir with or without ribavirin for HCV. N Engl J Med. 2014;370:1983-1992.

24. Pockros PJ, Reddy KR, Mantry PS, Cohen E, Bennett M, Sulkowski MS, et al. Efficacy of direct-acting antiviral combination for patients with hepatitis C virus genotype 1 infection and severe renal impairment or end-stage renal disease. Gastroenterology. 2016;150:1590-1598.

25. Lalezari J, Sullivan JG, Varunok P, Galen E, Kowdley KV, Rustgi V, et al. Ombitasvir/paritaprevir/r and dasabuvir plus ribavirin in HCV genotype 1-infected patients on methadone or buprenorphine. J Hepatol. 2015;63:364-369.

26. Feld JJ, Kowdley KV, Coakley E, Sigal S, Nelson DR, Crawford D, et al. Treatment of HCV with ABT-450/r-ombitasvir and dasabuvir with ribavirin. N Engl J Med. 2014;370:1594-1603.

27. Zeuzem S, Jacobson IM, Baykal T, Marinho RT, Poordad F, Bourliere M, et al. Retreatment of HCV with ABT-450/r-ombitasvir and dasabuvir with ribavirin. N Engl J Med. 2014;370:1604-1614.

28. Sulkowski M, Hezode C, Gerstoft J, Vierling JM, Mallolas J, Pol S, et al. Efficacy and safety of 8 weeks versus 12 weeks of treatment with grazoprevir (MK-5172) and elbasvir (MK-8742) with or without ribavirin in patients with hepatitis C virus genotype 1 mono-infection and HIV/hepatitis C virus co-infection (C-WORTHY): a randomised, open-label phase 2 trial. Lancet. 2015;385:1087-1097.

29. Lawitz E, Gane E, Pearlman B, Tam E, Ghesquiere W, Guyader D, et al. Efficacy and safety of 12 weeks versus 18 weeks of treatment with grazoprevir (MK-5172) and elbasvir (MK-8742) with or without ribavirin for hepatitis C virus genotype 1 infection in previously untreated patients with cirrhosis and patients with previous null response with or without cirrhosis (C-WORTHY): a randomised, open-label phase 2 trial. Lancet. 2015;385:1075-1086.

30. Poordad F, Lawitz E, Kowdley KV, Cohen DE, Podsadecki T, Siggelkow S, et al. Exploratory study of oral combination antiviral therapy for hepatitis C. N Engl J Med. 2013;368:45-53.

31. Manns M, Pol S, Jacobson IM, Marcellin P, Gordon SC, Peng CY, et al. All-oral daclatasvir plus asunaprevir for hepatitis C virus genotype 1b: a multinational, phase 3, multicohort study. Lancet. 2014;384:1597-1605.

32. Gane EJ, Stedman CA, Hyland RH, Ding X, Svarovskaia E, Subramanian GM, et al. Efficacy of nucleotide polymerase inhibitor sofosbuvir plus the NS5A inhibitor ledipasvir or the NS5B non-nucleoside inhibitor GS-9669 against HCV genotype 1 infection. Gastroenterology. 2014;146:736-743 e731.

33. Poordad F, Sievert W, Mollison L, Bennett M, Tse E, Brau N, et al. Fixed-dose combination therapy with daclatasvir, asunaprevir, and beclabuvir for noncirrhotic patients with HCV genotype 1 infection. J Amer Med Assoc. 2015;313:1728-1735.
