# Supplementary material for: Modelling how responsiveness to interferon improves interferon-free treatment of hepatitis C virus infection
Source: PLoS Comput Biol. 2018 Jul 12;14(7):e1006335. doi: 10.1371/journal.pcbi.1006335 (PMC6057683; doi:10.1371/journal.pcbi.1006335)
Supplement: S4 Table — The datasets in S1 Table that consider patients without distinction in cirrhosis are summarized. (DOCX) [file pcbi.1006335.s007.docx]

**S4 Table. Response to DAA-based treatments from studies that do not distinguish between patients with and without cirrhosis.** The datasets in S1 Table that consider patients without distinction in cirrhosis are summarized.

|  | **Regimen** | **Genotype** | **% SVR (N)** | | **P-value** | | **Ref.** |
| --- | --- | --- | --- | --- | --- | --- | --- |
|  |  |  | **Naïve** | **Null** | **χ^2^** | **Fisher** |  |
| IFN based | Telaprevir + PegIFNα/RBV | 1 | 75.4 (1272) | 32 (147) | 5.17×10^-28^ | 3.54×10^-25^ | [1] |
|  | Simeprevir + PegIFNα/RBV | 1 | 91.3 (150) | 52 (50) | 5.53×10^-10^ | 8.61×10^-9^ | [2, 3] |
|  | Simeprevir + PegIFNα/RBV | 4 | 82.9 (35) | 40 (40) | 1.57×10^-4^ | 1.71×10^-4^ | [4] |
| IFN free | Ledipasvir + sofosbuvir | 1 | 99.1 (214) | 91.8 (49) | 2.24×10^-3^ | 1.21×10^-2^ | [5, 6] |
|  | Ledipasvir + sofosbuvir + RBV | 1 | 97.2 (217) | 95.7 (46) | 5.70×10^-1^ | 6.32×10^-1^ | [5, 6] |
|  | Grazoprevir + elbasvir | 1,4,6 | 94.4 (517) | 91.8 (49) | 4.66×10^-1^ | 5.17×10^-1^ | [7-9] |
|  | Ombitasvir + paritaprevir/ritonavir | 1b | 95.2 (42) | 89.7 (58) | 3.10×10^-1^ | 4.62×10^-1^ | [10, 11] |
|  | Daclatasvir + simeprevir | 1b | 84.9 (53) | 95 (20) | 2.42×10^-1^ | 4.29×10^-1^ | [12] |
|  | Daclatasvir + simeprevir + RBV | 1b | 74.5 (51) | 69.6 (23) | 6.58×10^-1^ | 7.78×10^-1^ | [12] |

**S4 Table References**

1. Vertex. Incivek highlights of prescribing information. <http://pi.vrtx.com/files/uspi_telaprevir.pdf>. 2013.

2. Zeuzem S, Berg T, Gane E, Ferenci P, Foster GR, Fried MW, et al. Simeprevir increases rate of sustained virologic response among treatment-experienced patients with HCV genotype-1 infection: a phase IIb trial. Gastroenterology. 2014;146:430-441.

3. Wei L, Han T, Yang D, Heo J, Shang J, Cheng J, et al. Simeprevir plus peginterferon/ribavirin for HCV genotype 1-infected treatment-naive patients in China and South Korea. J Gastroenterol Hepatol. 2016;31:912-920.

4. Moreno C, Hezode C, Marcellin P, Bourgeois S, Francque S, Samuel D, et al. Efficacy and safety of simeprevir with PegIFN/ribavirin in naive or experienced patients infected with chronic HCV genotype 4. J Hepatol. 2015;62:1047-1055.

5. Afdhal N, Zeuzem S, Kwo P, Chojkier M, Gitlin N, Puoti M, et al. Ledipasvir and sofosbuvir for untreated HCV genotype 1 infection. N Engl J Med. 2014;370:1889-1898.

6. Afdhal N, Reddy KR, Nelson DR, Lawitz E, Gordon SC, Schiff E, et al. Ledipasvir and sofosbuvir for previously treated HCV genotype 1 infection. N Engl J Med. 2014;370:1483-1493.

7. Kwo P, Gane EJ, Peng CY, Pearlman B, Vierling JM, Serfaty L, et al. Effectiveness of elbasvir and grazoprevir combination, with or without ribavirin, for treatment-experienced patients with chronic hepatitis C infection. Gastroenterology. 2017;152:164-175 e164.

8. Dore GJ, Altice F, Litwin AH, Dalgard O, Gane EJ, Shibolet O, et al. Elbasvir-grazoprevir to treat hepatitis C virus infection in persons receiving opioid agonist therapy: A randomized trial. Ann Intern Med. 2016;165:625-634.

9. Zeuzem S, Ghalib R, Reddy KR, Pockros PJ, Ben Ari Z, Zhao Y, et al. Grazoprevir-elbasvir combination therapy for treatment-naive cirrhotic and noncirrhotic patients with chronic hepatitis C virus genotype 1, 4, or 6 infection: a randomized trial. Ann Intern Med. 2015;163:1-13.

10. Lawitz E, Makara M, Akarca US, Thuluvath PJ, Preotescu LL, Varunok P, et al. Efficacy and safety of ombitasvir, paritaprevir, and ritonavir in an open-label study of patients with genotype 1b chronic hepatitis C virus infection with and without cirrhosis. Gastroenterology. 2015;149:971-980 e971.

11. Chayama K, Notsumata K, Kurosaki M, Sato K, Rodrigues L, Jr., Setze C, et al. Randomized trial of interferon- and ribavirin-free ombitasvir/paritaprevir/ritonavir in treatment-experienced hepatitis C virus-infected patients. Hepatology. 2015;61:1523-1532.

12. Zeuzem S, Hezode C, Bronowicki JP, Loustaud-Ratti V, Gea F, Buti M, et al. Daclatasvir plus simeprevir with or without ribavirin for the treatment of chronic hepatitis C virus genotype 1 infection. J Hepatol. 2016;64:292-300.
