# Supplementary material for: Modelling how responsiveness to interferon improves interferon-free treatment of hepatitis C virus infection
Source: PLoS Comput Biol. 2018 Jul 12;14(7):e1006335. doi: 10.1371/journal.pcbi.1006335 (PMC6057683; doi:10.1371/journal.pcbi.1006335)
Supplement: S5 Table — Definitions of model parameters and their typical values employed. Variations are mentioned in the text. (DOCX) [file pcbi.1006335.s008.docx]

**S5 Table. Model parameters.** Definitions of model parameters and their typical values employed. Variations are mentioned in the text.

| **Symbol** | **Definition** | **Value** | **Ref.** |
| --- | --- | --- | --- |
| $d_{T}$ | Target cell death rate constant | 0.01 d^-1^ | [1] |
| $\beta$ | Infection rate constant | 3×10^-7^ ml virion^-1^ day^-1^ | [1] |
| $\eta_{1}$ | IFN efficacy in blocking infection of cells T_1_ | 0 | [2] |
| $\eta_{2}$, $\eta_{3}$ | IFN efficacy in blocking infection of cells T_2_,T_3_ | 1 | [2] |
| $\varepsilon_{1}$,$\varepsilon_{2}$ | IFN efficacy in blocking viral production from cells I_1,_I_2_ | 0 | [2] |
| $\varepsilon_{3}$ | IFN efficacy in blocking viral production from cells I_3_ | 1 | [2] |
| c | HCV clearance rate constant | 5.5 d^-1^ | [3] |
| $\delta$ | Infected cell death rate constant | 0.2 d^-1^ | [3] |
| $\mu$ | Mutation rate | 3×10^-5^ | [1, 4, 5] |
| $s$ | Target cell production rate | 1.3×10^5^ cells d^-1^ ml^-1^ | [1] |
| $p_{0}$ | Production rate of wild-type virions | 19 virions cell^-1^ d^-1^ | [1] |
| $p_{1}$ | Production rate of RAVs | $p_{0}\gamma$ |  |
| $\gamma$ | Pre-treatment relative fitness of a single mutant | Varied |  |
| $p_{00}$ | Production rate of wild-type virions when barrier is 2 | 19 virions cell^-1^ d^-1^ | [1] |
| $p_{10}$,$p_{01}$ | Production rate of single mutants when barrier is 2 | $\gamma p_{00}$ |  |
| $p_{11}$ | Production rate of double mutants when barrier is 2 | $\gamma^{2}p_{00}$ |  |
| $r_{T}$ | Target cell proliferation rate constant | 1.25 d^-1^ | [1, 6] |
| $r_{I}$ | Proliferation rate constant of infected cells | 0 .6 d^-1^ | [6] |
| $T_{\max}$ | Carrying capacity of liver for hepatocytes | 1.3 $\times$ 10^7^cells ml^-1^ | [1] |
| *N* | Non-target hepatocytes | 0.65 $\times$ 10^7^ cells ml^-1^ | [1] |
| $\varepsilon_{DAA}^{0}$ | DAA efficacy against wild type strain | Varied |  |
| $\varepsilon_{DAA}^{1}$ | DAA efficacy against RAV-carrying strain | Varied |  |
| $\varepsilon_{DAA}^{00}$ | DAA efficacy against wild type strain when barrier is 2 | Varied |  |
| $\varepsilon_{DAA}^{10}$,$\varepsilon_{DAA}^{01}$ | DAA efficacy against single mutant when barrier is 2 | Varied |  |
| $\varepsilon_{DAA}^{11}$ | DAA efficacy against double mutant when barrier is 2 | $\varepsilon_{DAA}^{11}=\frac{\varepsilon_{DAA}^{10}\times\varepsilon_{DAA}^{01}}{\varepsilon_{DAA}^{00}}$ |  |

**S5 Table References**

1. Rong L, Dahari H, Ribeiro RM, Perelson AS. Rapid emergence of protease inhibitor resistance in hepatitis C virus. Sci Transl Med. 2010;2:30ra32.

2. Padmanabhan P, Garaigorta U, Dixit NM. Emergent properties of the interferon-signalling network may underlie the success of hepatitis C treatment. Nat Commun. 2014;5:3872.

3. Dahari H, Layden-Almer JE, Kallwitz E, Ribeiro RM, Cotler SJ, Layden TJ, et al. A mathematical model of hepatitis C virus dynamics in patients with high baseline viral loads or advanced liver disease. Gastroenterology. 2009;136:1402-1409.

4. Ribeiro RM, Li H, Wang S, Stoddard MB, Learn GH, Korber BT, et al. Quantifying the diversification of hepatitis C virus (HCV) during primary infection: estimates of the in vivo mutation rate. PLoS Pathog. 2012;8:e1002881.

5. Powdrill MH, Tchesnokov EP, Kozak RA, Russell RS, Martin R, Svarovskaia ES, et al. Contribution of a mutational bias in hepatitis C virus replication to the genetic barrier in the development of drug resistance. Proc Natl Acad Sci USA. 2011;108:20509-20513.

6. Dahari H, Ribeiro RM, Perelson AS. Triphasic decline of hepatitis C virus RNA during antiviral therapy. Hepatology. 2007;46:16-21.
