# Supplementary material for: Modelling how responsiveness to interferon improves interferon-free treatment of hepatitis C virus infection
Source: PLoS Comput Biol. 2018 Jul 12;14(7):e1006335. doi: 10.1371/journal.pcbi.1006335 (PMC6057683; doi:10.1371/journal.pcbi.1006335)
Supplement: S1 Text — (DOCX) [file pcbi.1006335.s009.docx]

**S1 Text. Analytical approximation of the pre-treatment steady state with a single resistance locus**

We solved Eqs. (1)-(4) (Methods) for steady state, *i.e.*, by setting the left hand sides of all the equations to zero. When cell proliferation was negligible, the resulting equations became:

(S1.1)

$0=T_{i}(1-\eta_{i})\beta_{j}V_{j}-\delta I_{i}^{j}; i\in\{1,2,3\}, j\in\{0,1\}$ (S1.2)

$0=(1-\mu)p_{0}\sum_{i=1}^{3} (1-\varepsilon_{i})I_{i}^{0}-cV_{0}$ (S1.3)

$0=\mu p_{0}\sum_{i=1}^{3} (1-\varepsilon_{i})I_{i}^{0}+p_{1}\sum_{i=1}^{3} (1-\varepsilon_{i})I_{i}^{1}-cV_{1}$ (S1.4)

where we recognized that $\varepsilon_{DAA}^{j}=0$ pre-treatment. Using $I_{i}^{0}=T_{i}(1-\eta_{i})\beta_{0}V_{0}/\delta$ and $I_{i}^{1}=T_{i}(1-\eta_{i})\beta_{1}V_{1}/\delta$ from Eq. (S1.2) in Eqs. (S1.3) and (S1.4) yielded

$\left[\frac{(1-\mu)p_{0}\beta_{0}V_{0}}{\delta}\right]\sum_{i=1}^{3} (1-\varepsilon_{i})T_{i}(1-\eta_{i})=cV_{0}$ (S1.5)

and

$\left[\frac{\mu p_{0}\beta_{0}V_{0}}{\delta}+\frac{p_{1}\beta_{1}V_{1}}{\delta}\right]\sum_{i=1}^{3} (1-\varepsilon_{i})T_{i}(1-\eta_{i})=cV_{1}$. (S1.6)

Dividing Eq. (S1.6) by Eq. (S1.5) eliminated the term containing *T_i_* and yielded

$\frac{\mu p_{0}\beta_{0}V_{0}+p_{1}\beta_{1}V_{1}}{(1-\mu)p_{0}\beta_{0}V_{0}}=\frac{V_{1}}{V_{0}}$ (S1.7)

Recognizing that $\mu\ll1$ and rearranging terms yielded the relative mutant population, or frequency,

$\frac{V_{1}}{V_{0}}=\frac{\mu}{1-\frac{p_{1}\beta_{1}}{p_{0}\beta_{0}}}$. (S1.8)

The ratios *p*_1_/*p*_0_ and *β*_1_/*β*_0_ represent the reduction in replicative ability and infectivity, respectively, of the virus due to the resistance mutation. The two ratios together define the overall loss of viral fitness due to the mutation. If this overall loss is quantified using a selective disadvantage, $1-\gamma$, so that $\frac{p_{1}\beta_{1}}{p_{0}\beta_{0}}=\gamma$, Eq. (S1.8) reduces to the classical mutation-selection balance, $\frac{V_{1}}{V_{0}}=\frac{\mu}{1-\gamma}$, well-known in population genetics [1] and consistent with earlier models of HIV and HCV drug resistance [2-6].

Importantly, the mutant frequency was independent of the distribution of cells into the three IFN response phenotypes. The absolute viral population, however, did depend on the latter distribution. To demonstrate this, we considered Eqs. (S1.1)-(S1.4) again with the recognition that $V_{1}\ll V_{0}$ pre-treatment. Combining Eqs. (S1.1)-(S1.3) to eliminate *T_i_* and $I_{i}^{0}$ yielded an equation for *V*_0_:

(S1.9)

IFN activity blocked *de novo* infection of cells *T*_2_ and *T*_3_ and viral production from cells *I*_3_. Cells *T*_1_, *I*_1_, and *I*_2_ were refractory to IFN activity. Consequently, the effectiveness terms assumed the values *ε*_1_=*ε*_2_=0, *ε*_3_=1, *η*_1_=0, and *η*_2_=*η*_3_=1. Using these values in Eq. (S1.9) and rearranging terms yielded

(S1.10)

and hence

. (S1.11)

**S1 Text References**

1. Hartl DL, Clark AG. Principles of Population Genetics. Fourth ed. Sunderland, MA: Sinauer Associates, Inc.; 2007.

2. Nowak MA, May RM. Virus Dynamics: Mathematical Principles of Immunology and Virology. New York: Oxford University Press; 2000.

3. Ribeiro RM, Bonhoeffer S, Nowak MA. The frequency of resistant mutant virus before antiviral therapy. AIDS. 1998;12:461-465.

4. Gadhamsetty S, Dixit NM. Estimating frequencies of minority nevirapine-resistant strains in chronically HIV-1-infected individuals naive to nevirapine by using stochastic simulations and a mathematical model. J Virol. 2010;84:10230-10240.

5. Perelson AS, Guedj J. Modelling hepatitis C therapy-predicting effects of treatment. Nature reviews. 2015;12:437-445.

6. Rong L, Dahari H, Ribeiro RM, Perelson AS. Rapid emergence of protease inhibitor resistance in hepatitis C virus. Sci Transl Med. 2010;2:30ra32.
