# Supplementary material for: Modelling how responsiveness to interferon improves interferon-free treatment of hepatitis C virus infection
Source: PLoS Comput Biol. 2018 Jul 12;14(7):e1006335. doi: 10.1371/journal.pcbi.1006335 (PMC6057683; doi:10.1371/journal.pcbi.1006335)
Supplement: S2 Text — (DOCX) [file pcbi.1006335.s010.docx]

**S2 Text. Model formulation with multiple resistance loci**

We extended the model in Eqs. (1)-(4) (Methods) to include multiple loci where mutations contribute to the development of resistance. We distinguished viral strains by the combinations of mutations at these loci that the strains contained. Let B be the number of such loci involved. B thus denotes the genetic barrier of the treatment. Depending on whether each locus has a resistance associated mutation or not, a total of *S*=2*^B^* distinct viral variants can exist, including the wild-type, which contains no mutations. We numbered these variants with the index *j*, where $j\in\left\{0,1,2,...,S-1\right\}$. The following equations then described the ensuing dynamics.

(S2.1)

$\frac{dI_{i}^{j}}{dt}=r_{I}I_{i}^{j}\left[1-\frac{\sum_{i=1}^{3} \left(T_{i}+\sum_{j=0}^{S-1} I_{i}^{j}\right)+N}{T_{\max}}\right]+T_{i}(1-\eta_{i})\beta_{j}V_{j}-\delta I_{i}^{j}; i\in\{1,2,3\}, j\in\{0,1,2,...,S-1\}$ (S2.2)

$\frac{dV_{j}}{dt}=\sum_{h=0}^{S-1} \sum_{i=1}^{3} (1-\varepsilon_{DAA}^{h})M_{jh}p_{h}(1-\varepsilon_{i})I_{i}^{h}-cV_{j}; j,h\in\{0,1,2,...,S-1\}$ (S2.3)

where M*_jh_* is the probability that strain *j* is produced by mutation of strain *h* and is given by

$M_{jh}=\mu^{d(j,h)}\left(1-\mu\right)^{B-d(j,h)}$, (S2.4)

where *d*(*j*,*h*) is the Hamming distance between the two strains and *µ* is the mutation rate. The other terms in Eqs. (S2.1)-(S2.4) have the same meanings as in Eqs. (1)-(4).
