# Supplementary material for: Modelling how responsiveness to interferon improves interferon-free treatment of hepatitis C virus infection
Source: PLoS Comput Biol. 2018 Jul 12;14(7):e1006335. doi: 10.1371/journal.pcbi.1006335 (PMC6057683; doi:10.1371/journal.pcbi.1006335)
Supplement: S3 Text — (DOCX) [file pcbi.1006335.s011.docx]

**S3 Text. Analytical approximation of the pre-treatment steady state with two or more resistance loci**

***Two loci***

With two resistance loci, four distinct types of viral genomes, defined by whether each locus carried a mutation or not, emerged. We denoted these genomes using $j\in\left\{00,01,10,11\right\}$, where 0 represented the wild-type allele and 1 a mutation at any given locus. Eqs. (S2.1)-(S2.4) with the above definition of *j* then yielded the corresponding dynamics. The pre-treatment steady state was again obtained by setting the left-hand sides of the equations to zero. Neglecting cell proliferation and back mutations and recognizing that $V_{00}\gg V_{01},V_{10}\gg V_{11}$ yielded

(S3.1)

$0=T_{i}(1-\eta_{i})\beta_{j}V_{j}-\delta I_{i}^{j}; i\in\{1,2,3\}, j\in\left\{00,01,10,11\right\}$ (S3.2)

$0=\sum_{i=1}^{3} \left(1-\mu\right)^{2}p_{00}(1-\varepsilon_{i})I_{i}^{00}-cV_{00}$ (S3.3)

$0=\sum_{i=1}^{3} \left(1-\mu\right)^{2}p_{01}(1-\varepsilon_{i})I_{i}^{01}+\sum_{i=1}^{3} \mu\left(1-\mu\right)p_{00}(1-\varepsilon_{i})I_{i}^{00}-cV_{01}$ (S3.4)

$0=\sum_{i=1}^{3} \left(1-\mu\right)^{2}p_{10}(1-\varepsilon_{i})I_{i}^{10}+\sum_{i=1}^{3} \mu\left(1-\mu\right)p_{00}(1-\varepsilon_{i})I_{i}^{00}-cV_{10}$ (S3.5)

$0=\sum_{i=1}^{3} \left(1-\mu\right)^{2}p_{11}(1-\varepsilon_{i})I_{i}^{11}+\sum_{i=1}^{3} \mu\left(1-\mu\right)p_{10}(1-\varepsilon_{i})I_{i}^{10}+\sum_{i=1}^{3} \mu\left(1-\mu\right)p_{01}(1-\varepsilon_{i})I_{i}^{01}-cV_{11}$ (S3.6)

Further, using $\mu\ll1$ and the effectiveness values defined above, we obtained

(S3.7)

$\frac{V_{01}}{V_{00}}\approx\frac{\mu}{1-\gamma_{01}}; \frac{V_{10}}{V_{00}}\approx\frac{\mu}{1-\gamma_{10}}; \frac{V_{11}}{V_{00}}\approx\frac{\mu^{2}}{1-\gamma_{11}}\left[1+\frac{\gamma_{10}}{1-\gamma_{10}}+\frac{\gamma_{01}}{1-\gamma_{01}}\right]$ (S3.8)

where $\gamma_{01}=\frac{p_{01}\beta_{01}}{p_{00}\beta_{00}}$, $\gamma_{10}=\frac{p_{10}\beta_{10}}{p_{00}\beta_{00}}$, and $\gamma_{11}=\frac{p_{11}\beta_{11}}{p_{00}\beta_{00}}$.

***Multiple loci***

We extended the above formalism to the scenario where *n* loci were involved in drug resistance. For ease of notation, we denote wild-type virions with the index ‘0’; single mutants with index $j\in\left\{1,2,3,...,n\right\}$ denoting the position where the mutation occurs; double mutants with $jk$, where $j,k\in\left\{1,2,3,...,n\right\}$ and $j<k$; triple mutants with $jkl$, where $j,k,l\in\left\{1,2,3,...,n\right\}$ and $j<k<l$; and so on. We also assumed for simplicity that RAVs influenced viral production rates and not infectivity, so that $\beta_{j}=\beta_{jk}=\beta_{jkl}=...=\beta$. At steady state, following the above arguments, we wrote the following balances on cell types,

(S3.9)

$0=T_{i}(1-\eta_{i})\beta V_{j}-\delta I_{i}^{j}$ (S3.10)

$0=T_{i}(1-\eta_{i})\beta V_{jk}-\delta I_{i}^{jk}$ (S3.11)

$0=T_{i}(1-\eta_{i})\beta V_{jkl}-\delta I_{i}^{jkl}$ (S3.12)

$0=T_{i}(1-\eta_{i})\beta V_{jkl...(n terms)}-\delta I_{i}^{jkl...(n terms)}$ (S3.13)

and on viral populations,

$0=\sum_{i=1}^{3} \left(1-\mu\right)^{n}p_{0}(1-\varepsilon_{i})I_{i}^{0}-cV_{0}$ (S3.14)

$0=\sum_{i=1}^{3} \left(1-\mu\right)^{n-1}p_{j}(1-\varepsilon_{i})I_{i}^{j}+\sum_{i=1}^{3} \mu\left(1-\mu\right)^{n-1}p_{0}(1-\varepsilon_{i})I_{i}^{0}-cV_{j}$ (S3.15)

$\begin{matrix} 0=\sum_{i=1}^{3} \left(1-\mu\right)^{n-2}p_{jk}(1-\varepsilon_{i})I_{i}^{jk}+\sum_{i=1}^{3} \mu\left(1-\mu\right)^{n-2}(1-\varepsilon_{i})\left(p_{j}I_{i}^{j}+p_{k}I_{i}^{k}\right) \\ +\sum_{i=1}^{3} \mu^{2}\left(1-\mu\right)^{n-2}p_{0}(1-\varepsilon_{i})I_{i}^{0}-cV_{jk} \end{matrix}$ (S3.16)

$\begin{matrix} 0=\sum_{i=1}^{3} p_{jkl...(n terms)}(1-\varepsilon_{i})I_{i}^{jkl...(n terms)}+...+\sum_{i=1}^{3} \mu^{n-2}(1-\varepsilon_{i})\left(\sum_{j,k} p_{jk}I_{i}^{jk}\right) \\ +\sum_{i=1}^{3} \mu^{n-1}(1-\varepsilon_{i})\left(\sum_{j} p_{j}I_{i}^{j}\right)+\sum_{i=1}^{3} \mu^{n}p_{0}(1-\varepsilon_{i})I_{i}^{0}-cV_{jkl...(n terms)} \end{matrix}$ (S3.17)

In the above equations, $i\in\{1,2,3\}$ and $j,k,l,...\in\left\{1,2,3,...,n\right\}$ and $j<k<l<...$ Further, the efficacy terms were identical to those mentioned earlier: *ε*_1_=*ε*_2_=0, *ε*_3_=1, *η*_1_=0, and *η*_2_=*η*_3_=1. To solve these equations, we followed the procedure outlined above. Eliminating $I_{i}^{0}$ from Eqs. (S3.10) and (S3.14) yielded,

$\sum_{i=1}^{3} (1-\varepsilon_{i})T_{i}(1-\eta_{i})=\frac{c\delta}{p_{0}\beta\left(1-\mu\right)^{n}}$. (S3.18)

Similarly, substituting $I_{i}^{0}$ and $I_{i}^{j}$ from Eqs. (S3.10) in (S3.15) and rearranging yielded,

$\frac{V_{j}}{V_{0}}=\frac{\sum_{i=1}^{3} \mu\left(1-\mu\right)^{n-1}p_{0}(1-\varepsilon_{i})T_{i}(1-\eta_{i})\frac{\beta}{c\delta}}{1-\sum_{i=1}^{3} \left(1-\mu\right)^{n-1}p_{j}(1-\varepsilon_{i})T_{i}(1-\eta_{i})\frac{\beta}{c\delta}}\approx\frac{\mu}{1-\frac{p_{j}}{p_{0}}}$ (S3.19)

where the latter simplification followed from using Eq. (S3.18) and letting $\mu\ll1$. Proceeding in this way, we obtained recursive relations for double mutants,

$\frac{V_{jk}}{V_{0}}\approx\frac{\mu^{2}+\mu\left(\frac{p_{j}}{p_{0}}\frac{V_{j}}{V_{0}}+\frac{p_{k}}{p_{0}}\frac{V_{k}}{V_{0}}\right)}{1-\frac{p_{jk}}{p_{0}}}$ (S3.20)

and all higher mutants,

$\frac{V_{jkl...(n terms)}}{V_{0}}\approx\frac{\mu^{n}+\mu^{n-1}\sum_{j} \frac{p_{j}}{p_{0}}\frac{V_{j}}{V_{0}}+\mu^{n-2}\sum_{j,k} \frac{p_{jk}}{p_{0}}\frac{V_{jk}}{V_{0}}+...+\mu\sum_{j,k,l,...(n-1 terms)} \frac{p_{jkl...(n-1 terms)}}{p_{0}}\frac{V_{jkl...(n-1 terms)}}{V_{0}}}{1-\frac{p_{jkl...(n terms)}}{p_{0}}}$.

(S3.21)

The productivities $p_{j},p_{jk},p_{jkl},...$ defined the fitness of different single, double, triple, etc. mutants. These fitness values are difficult to obtain [1]. Only recently have the fitness of all possible single mutants in the NS5A region of the HCV genome been experimentally identified [2]. The fitness of pairs and higher mutants remained unknown. Empirical fitness models are being developed using ideas from statistical mechanics [3]. As a simplification, the fitness is often assumed to depend on the number of RAVs and not on the specific combination of RAVs. Such an approximation has been found to be reasonable with HIV [4, 5]. Further, if epistatic interactions between mutations are neglected so that each mutation carries a fixed fitness penalty, the relative fitness of genomes carrying *m* RAVs may be written as $\gamma^{m}$. Eq. (S3.21) then simplified to

$\frac{V_{jkl...(n terms)}}{V_{0}}\approx\frac{\mu^{n}+n\mu^{n-1}\gamma\frac{V_{j}}{V_{0}}+\left(\begin{matrix} n \\ 2 \end{matrix}\right)\mu^{n-2}\gamma^{2}\frac{V_{jk}}{V_{0}}+...+\left(\begin{matrix} n \\ n-1 \end{matrix}\right)\mu\gamma^{n-1}\frac{V_{jkl...(n-1 terms)}}{V_{0}}}{1-\gamma^{n}}$ (S3.22)

where $V_{j}$ was the population of any single mutant, $V_{jk}$ that of any double mutant, and so on. Note that the populations of all single mutants were equal in this scenario; similarly, the populations of all double mutants were equal; and so on. Further, we recognized following the above derivation that , yielding the dependence of the viral population on IFN-refractoriness,

. (S3.23)

**S3 Text References**

1. Perales C, Quer J, Gregori J, Esteban J, Domingo E. Resistance of hepatitis C virus to inhibitors: complexity and clinical implications. Viruses. 2015;7:2902.

2. Qi H, Olson CA, Wu NC, Ke R, Loverdo C, Chu V, et al. A quantitative high-resolution genetic profile rapidly identifies sequence determinants of hepatitis C viral fitness and drug sensitivity. PLoS Pathog. 2014;10:e1004064.

3. Hart GR, Ferguson AL. Empirical fitness models for hepatitis C virus immunogen design. Phys Biol. 2015;12:066006.

4. Bonhoeffer S, Chappey C, Parkin NT, Whitcomb JM, Petropoulos CJ. Evidence for positive epistasis in HIV-1. Science. 2004;306:1547-1550.

5. Tripathi K, Balagam R, Vishnoi NK, Dixit NM. Stochastic simulations suggest that HIV-1 survives close to its error threshold. PLoS Comput Biol. 2012;8:e1002684.
